# The effect of foliar spraying of silver and iron nanoparticles as fertilizers on the quantity, quality, and antimicrobial properties of *Melissa officinalis* L. essential oil

**DOI:** 10.1371/journal.pone.0323296

**Published:** 2025-06-05

**Authors:** Faezeh Nabizadeh Samani, Mansureh Ghavam, Ali Tavili, Rouhollah Mirzaei

**Affiliations:** 1 Department of Nature Engineering, Faculty of Natural Resources and Earth Sciences, University of Kashan, Kashan, Iran; 2 Department of Reclamation of Arid and Mountainous Regions, Faculty of Natural Resources, University of Tehran, Karaj, Iran; 3 Department of Environment, Faculty of Natural Resources and Earth Sciences, University of Kashan, Kashan, Iran; National Institute of Agricultural Research - INRA, MOROCCO

## Abstract

*Melissa officinalis* L*.* as a medicinal plant used in traditional medicine to treat headaches caused by stress, anemia, nausea, dizziness, indigestion, colic, epilepsy, hysteria, cancer, and heart failure. The present study aimed to evaluate the effect of foliar spraying of silver and iron nanoparticles as fertilizers on yield, type, chemical compounds, and antimicrobial properties of *M. officinalis* leaf essential oil. For this purpose, plant cultivation was conducted in May 2020 as a completely random design. After one year of establishment, foliar spraying was conducted at the beginning of flowering, the peak of flowering, and the end of flowering at the levels of 20, 40, 60, 80, and 100 mg/L, and the control of iron and silver nanoparticles. After collecting and drying the leaves, the essential oil was extracted by water distillation, and the compounds were identified by Gas chromatography–mass spectrometry. The antibacterial activity of essential oils was evaluated by using agar diffusion method, Minimum growth inhibitory concentration, and Minimum concentration of bacterial lethality. The results showed that foliar spraying with different nano iron and silver treatments had a significant effect on the yield, percentage of compounds, and antimicrobial activity of *M. officinalis* leaf essential oil (p ≤ 0.01). The 60 mg/L of FeNPs concentration with 1.70% (w/w) had the highest yield of *M. officinalis* leaf essential oil. The neral (33.5–0%), citral (28.53–0%), geranial (28.25–0%), caryophyllene (20.71–0%), caryophyllene-oxide (19.73–7.36%), and geranial acetate (5.99–11.84%) were the dominant compounds of essential oil. The results of antimicrobial test showed that the lowest MIC value belongs to the treatments of nano iron 100, nano silver 20, and nano silver 60 mg/L with a value of <62.5 μg/mL. against *Pseudomonas aeruginosa*, that it was one times weaker than rifampin and three times more potent than the control treatment. However, the best treatment in terms of of the essential oil efficiency was for the samples sprayed with nanoiron 60 mg/L. In terms of the essential oil componds, was for the nanoiron treatment 100 mg/L with the predominance of caryophyllene oxide and geranyl acetate, and in terms of the antimicrobial activity, was for the nano silver treatment, 60 mg/L. Therefore, they can be promising and potential natural options for the production of *M. officinalis* essential oil under foliar spraying with economical and environmentally friendly fertilizers for consumption in various pharmaceutical and cosmetic industries.

## 1 Introduction

The cultivation and processing of medicinal plants is cost-effective when the desired level of secondary metabolites is observed in them. Therefore, appropriate environmental and nutritional factors should be selected for the development and cultivation of medicinal plants [1]. The ecological factors of the plant growth and cultivation environment are among the most critical factors affecting the quantity and quality of effective compounds in medicinal plants [2].

During the growth stages of medicinal plants, the correct consumption of nutrients plays an essential role in increasing their performance. During the growth stages of medicinal plants, the correct consumption of nutrients plays an essential role in increasing their performance and is effective in the quantity and quality of the effective ingredients of the produced product. Micronutrients are involved in many biochemical reactions of plants and due to their positive effect in increasing the quantitative and qualitative performance of medicinal plants, they are needed in the natural growth of plants [3]. Nevertheless, on the other hand, soil structure change, soil permeability reduction, groundwater pollution, nitrate accumulation, and heavy element toxicity are among the problems of excessive use of chemical fertilizers [4,5]. Nano compounds cause the gradual and controlled release of nutrients in the soil, and if replaced with conventional fertilizers, the frequency of fertilizer application will be reduced and as a result, the negative effects of excessive fertilizer consumption and soil toxicity will be minimized [6].

Today, the use of nanotechnology is expanding in all fields, including agriculture. If compounds with desirable properties such as effective concentration, proper solubility, stability, and high effectiveness are used, nano compounds improve plant nutrition [7]. If the micronutrient elements are mixed with the soil in the form of mineral compounds, by stabilizing these elements in the soil, the absorption efficiency will be reduced and it will not be economical. For this reason, alternative methods such as foliar spraying are used. Micronutrient elements play an essential role in cell differentiation, growth, and cell wall strength, and in most cases, the plant becomes resistant to pests and diseases. Therefore, insufficient absorption of elements leads to a decrease in the quality of the product. In this situation, foliar spraying is more economical and effective [8]. Since iron is part of the catalytic group of many oxidation and reduction enzymes, plants need iron the most among all micronutrients [9]. Iron plays a role in the structure of electron transporters such as cytochrome, which is required for photosynthesis, and also in respiration and biological fixation of nitrogen [10]. Despite the abundance of iron in the earth’s crust, it creates the greatest limitation in the production of agricultural products. The main cause of iron deficiency in calcareous soils is bicarbonate ion, and other factors of iron deficiency in the soil include soil alkalinity, lack of organic matter, heavy irrigation, and poor soil ventilation [8]. Studies have shown that the consumption of nano iron chelate affects the amount of essential oil and its compounds [11]. The review of studies indicated the improvement of quantitative and qualitative traits due to foliar application of iron fertilizer in various medicinal plants such as anise, peppermint and German chamomile [12–14]. It has also been proven that the amount of essential oil in black seed, rhododendron, and basil plants increased with the use of iron fertilizer [15,16]. On the other hand, studies show that foliar application of iron and zinc nanofertilizers significantly increased the biological performance of medicinal plants [17].

*Melissa officinalis* L., with the Persian name of “Baderanjbooieh”, is a perennial plant belonging to the Lamiaceae family, the Dicotyledons, and the Lipaceae order [18]. The height of this plant is 30–80 cm and it has heart-shaped leaves, serrated and covered with webs, which are dark green on the upper surface and light green on the lower surface. Flowers appear from June to mid-August and are white or pink [19].

*M. officinalis* contains essential oil, phenolic acids, flavonoids, triterpenes, and tannins [20]. Studies have shown that the most important component of *M. officinalis* essential oil is citronellol (20–50%). Other compounds of this essential oil include citral, geraniol, linalool, and beta caryophyllene [21]. *M. officinalis* has the therapeutic effects of calming, anti-depressant, hypnotic, pain reliever, heart tonic, diuretic, antispasmodic, carminative, stomachic, diuretic, memory enhancer, antidote, menstrual stimulant, blood pressure reducer and antiseptic [20]. In traditional medicine for centuries, *M. officinalis* has been used to treat stress headaches, nervousness, anemia, nausea, dizziness, depression, syncope, boredom, indigestion, colic, ulcers, epilepsy, psychosis, hysteria, cancer, and heart failure [20,22,23]. The biological properties of *M. officinalis* include antioxidant [24], anticancer [25], anti-inflammatory [26] and It is antimicrobial [27] In modern pharmacology, M. officinalis has various uses for treating diseases such as migraine, cancer control, Alzheimer’s, and rheumatism [28].

Therefore, the present study study aims to 1) investigate the effectiveness of using two types of nano-silver and iron fertilizers simultaneously on the essential oil of M. officinalis; 2) measure the quality and quantity of the extracted essential oil under different spray treatments; and 3) evaluate the potency and potential of the essential oil in destroying some microbial strains.

## 2 Materials and methods

### 2.1 Cultivation

All methods conducted comply with relevant institutional, national, and international guidelines and legislation. The *M. officinalis* planting area was selected in one of the fields of Saman city (with longitude 50° 53’ and latitude 32° 27’ and altitude 2020 meters above sea level) with a distance of 2 km from the city. Saman region has a cold steppe climate based on Gosen’s classification, and a temperate and cold climate with hot and dry summers based on Kopen’s classification [29].

In the spring and early May, the land was prepared by plowing with a tractor, disking, leveling, creating a ridge and ridge, and dividing the plots with a shovel and manually. After preparing the substrate, one day before planting, animal manure was added to the intended plots and then mixed with the soil. Before irrigation, potassium, and urea chemical fertilizers were added to the plants by surface spreading by hand. According to the number of treatments and repetitions, the farm was divided into plots. In the division of plots for foliar application and the existence of different growth conditions in the region, it was tried so that similar plots are not next to each other and each treatment is distributed in different parts of the land. For each level of foliar spraying with nanoparticles, 4 repetitions and 5 plots were also used as controls (no foliar spraying) and in total, the total number of experimental plots consisted of 45 plots with dimensions of 1 m × 1 m, which were demarcated using wood, thread, and stones.

Cultivation was done by manual transplanting in late May and early June of 2019. In order to plant seedlings in each plot, 2 planting rows were considered with a distance of 50 cm between the rows. During all the steps, the irrigation of the field was done according to the moisture status of the soil and the environmental conditions with an interval of 12 days and in the form of flooding in the agricultural land.

After the establishment of one-year plants, iron/silver nanoparticle foliar spraying during the growth stages of *M. officinalis* was carried out at three times: the beginning of flowering, the peak of flowering, and the end of flowering, two weeks apart from each other, in a completely randomized design with 4 replications. Levels of 20, 40, 60, 80, and 100 mg/L of iron nanoparticles, and levels of 20, 40, 60, 80, and 100 mg/L of silver nanoparticles were selected for foliar spraying. After the flowering stage, the flowering branches of the plants were collected by gardening shears and dried in the shade.

### 2.2 Laboratory operations

#### 2.2.1 Essential oil extraction and yield calculation.

After transferring the dried samples to the laboratory and to extract the essential oil by steam distillation, 100 g of the flowering branch and stem of *M. officinalis* were weighed and after grinding using an electric mill, they were placed in the Clevenger and allowed to extract the essential oil for 2–3 hours. After that, essential oils were poured into special containers and water was extracted with the help of sodium sulfate. Then, the essential oils were kept away from light at a temperature of 4°C until injection into the mass spectrometer and antimicrobial test. The amount of essential oil was weighed using a sensitive scale. The yield of essential oil (quantity) was calculated based on the dry weight of the sample and reported as mean ± standard deviation. The percentage of essential oil was calculated using the equation [30] (1):


yieldofessentialoil=(weightofessentialoil/weightofdryplant)x100 (1)

#### 2.2.2 Analysis of the chemical composition of essential oils.

To identify the components of the essential oil, a chromatograph model 6890 coupled with a mass spectrometer model N-5973 manufactured by Agilent company with a HP-5MS capillary column with a stationary phase of methylphenylsiloxane 5% (length 30 meters, inner diameter 0.25 mm, layer thickness 0.25 μm) and ionization energy of 70 electron volts was used. Temperature planning for the analysis, at first, the oven temperature started at 60°C and then increased at a rate of 3°C/min to 246°C. The temperature of the injector and detector was 250 °C, the injected sample volume was 1μ with a split of 1.50, and helium carrier gas with a flow rate of 1.5 mL/min.

The chemical components of essential oils were identified based on the analysis of the chromatograms of each essential oil sample concerning the retention (inhibition) indices (RI) in relation to the standards of (C8-C20) n-alkane mixtures and the mass spectral data of each peak using Wiley-14 and NIST-14 spectral library, and comparing the results with literature [31].

#### 2.2.3 Antimicrobial activity.

In the antimicrobial activity test, clinical strains including Gram-positive bacteria *Staphylococcus aureus* and *Staphylococcus epidermidis* and Gram-negative bacteria *pseudomonas aeruginosa* were used, which were obtained from Iran Scientific and Technological Research Organization (IROST).

**2.2.3.1 Agar diffusion method:** The agar diffusion method was performed according to CLSI standards (CLSI, 2012). For this purpose, plates containing Mueller Hinton agar culture medium were prepared, wells with a diameter of 0.6 mm were created on the culture medium, and then the culture process was conducted using 0.10 µL of bacterial suspensions with turbidity equal to half McFarland in uniform conditions on the surface of the culture medium. The plant essential oil was dissolved in dimethylsulfoxide and reached a concentration of 0.30 mg/mL. The amount of 0.10 µL equivalent to 0.60 µg per well of essential oil was poured into the wells, the plates were placed in a 37°C incubator for 24 h, and its antimicrobial activity was determined for each microorganism by measuring the halo of non-growth [32].

**2.2.3.2 Minimum growth inhibitory concentration (MIC):** The minimum growth inhibitory concentration for microorganisms sensitive to essential oil/silver nanoparticles was calculated by the microdilution method (CLSI, 2012). For this purpose, sterile 96-well microplates were prepared. 95 µL of culture medium, 5 µL of bacterial suspension with a dilution of 0.5 McFarland, and 100 µL of different dilutions of the extract were added to each of the plates, and then the plate was heated in an incubator at a temperature of 37° C for 24 hours. According to the color change and turbidity of each microplate well, MIC or minimum growth inhibitory concentration was determined. The experiment was repeated three times for each sample. The average of the lowest concentrations that inhibited bacterial growth was reported as MIC [32].

**2.2.3.3 Minimum concentration of bacterial lethality (MBC):** To determine the minimum bacterial lethal concentration test, after 24 h of heating, 5 µL from each of the microplate wells in which there was no growth were inoculated into nutrient agar medium and heated for 24 h at 37°C. Colony-forming units were counted after incubation. MBC was the minimum concentration that could effectively reduce the growth of bacteria by 99.5% [32].

### 2.3 Statistical analysis

Statistical analysis was done with SPSS 22 software. First, the normality of the statistical variables was investigated through the Kolmogorov-Smirnov test, and one-way ANOVA analysis was used after checking the reliability of the data’s normality. Then, using Duncan’s post hoc test at a significance level of 1%, the difference between the average values of the data was evaluated. All data were expressed as mean ± standard deviation.

## 3 Results and discussion

### 3.1 Essential oil yield

The color of *M. officinalis* leaf essential oil under different treatments was deep yellow [10]. The results of analysis of variance showed that the effect of foliar spraying with different levels of silver and iron nanoparticles had a significant effect on the yield of Lemon balm leaf essential oil (p ≤ 0.01) (Table 1). The highest yield of *M. officinalis* leaf essential oil was observed in samples sprayed with iron 60 mg/L (1.70%). Various previous studies have confirmed the effect of foliar spraying with iron on increasing the yield of essential oils of different medicinal plants such as *Rosa × damascena* Herrm [33]; *Thymus vulgaris* L. [34]; *Mentha piperita* L. [35]. Liquid fertilizers spraying on plant leaves, the fertilizer is directly absorbed by plant tissues and organs [33]. Nanoparticles enter the plant on the surface of the leaf through the stomatal pores or the base of hairs and then are transferred to different tissues [36]. Iron is one of the essential, low-use, and immobile elements for plants. This element is part of the catalytic group of many oxidation and regeneration enzymes and is required for the production of chlorophyll [35]. Iron element increases the amount of chlorophyll production and chloroplast growth, and consequently increases the amount of photosynthesis and carbohydrate production, and as a result, provides better conditions for plant growth and development [37]. It seems that due to the effect of iron and zinc elements on the growth and development of the plant, one of the reasons for the increase in the amount of essential oil is the increase in the photosynthetic activity of the plant and the role of this element in the activity of the chloroplast structure, which leads to the production of more essential oil-producing cells in the leaf [38]. Due to their small size and high solubility, nano-iron compounds are absorbed faster by plants, and therefore, by using these materials, optimal conditions for plant growth are created [39]. Essential oils with terpene origin have an urgent need for NADH and ATP in their constituent units such as dimethyl pyrophosphate and isopentyl pyrophosphate. The production of essential oil in plants is directly related to photosynthesis and the production of photosynthetic products, and the existing correlation between essential oil and photosynthesis in the synthesis of essential oil, especially monoterpenes, and for providing NADH and ATP, glucose acts as a suitable precursor. The necessary substrate for providing energy and synthesizing effective compounds in essential oil is glucose obtained from photosynthesis. With the increase of iron in the plant, the power of photosynthesis and the precursors of phenolic compounds required for the synthesis of essential oils increase, and as a result, the result of essential oil production also increases [40].

**Table 1:** Yield of *M. officinalis* leaf essential oil under the influence of foliar spraying with different levels of silver and iron nanoparticles.

	Treatment (mg/L)	Mean±SD
FeNPs	20	0.80^b ^± 0.05
	40	0.13^f ^± 0.05
	60	1.70^a ^± 0.08
	80	0.36 ^e ^± 0.06
	100	0.05^h ^± 0.01
AgNps	20	0.40^d ^± 0.02
	40	0.70^c ^± 0.03
	60	0.16^f ^± 0.05
	80	0.73^c ^± 0.03
	100	0.74^c ^± 0.04
Control	0	0.07^g ^± 0.01

٭Different letters indicate statistically significant differences (P ≤ 0.01).

On the other hand, the results showed that the yield of the essential oil of the samples sprayed with nano-iron treatments had a decreasing trend from the concentration of 80 mg/L. So the samples treated with 100 mg/L of nano-iron had the lowest essential oil yield (0.5%) among all the studied treatments. It seems that the increase of iron element has a decreasing effect on the yield of essential oil.

Also, the results indicated that increasing the concentration of silver nanoparticle foliar spraying treatments (except for the 60 mg/L) increased the yield of *M. officinalis* leaf essential oil compared to the control treatment [41] in the study of the effect of silver nanoparticles on *M. officinalis* found that silver nanoparticle treatment of 60 mg/L had the greatest effect on plant growth, which is contrary to the present results [42] found that in *Eichhornia crassipes* (Mart) solms., silver nanoparticles in concentrations of 1 and 100 mg/L caused an increase in growth, while the concentration of 10 mg/L caused a decrease in plant growth; Therefore, the effects of silver nanoparticles on growth are different depending on the concentration of nanoparticles and the type of plant species. Silver nanoparticles improve the biosynthesis of secondary metabolites by inducing the defense system [43]. Several reports show that silver nanoparticles may have negative or positive effects on the growth of higher plants [44].

Based on the results, the yield of *M. Officinalis* leaf essential oil without treatment (control) was 0.07% and after foliar treatment with iron, it was 100 mg/L. In previous studies, the yield of *M. Officinalis* essential oil from the south of Marivan city was 1.18% [45], from Serbia 0.18% [46] and 0.470% from Algiers [27]which are more than the value of the present study.

### 3.2 Chemical compounds of essential oil

The results of the analysis of *M. officinalis* leaf essential oil under different foliar spraying treatments with different levels of iron and silver nanoparticles showed that there was a significant difference between the number and relative percentage of compounds with the essential oil sample extracted from the control plots (p ≤ 0.01) (S1–S11 Fig in S1 File; S1–S11 Tables in S1 File). The highest number of compounds was observed under foliar treatment with nano silver 20 and 100 mg/L (48 compounds; with a relative percentage of 98.98% and 100%; respectively) and the lowest number of compounds was observed in the foliar treatment with iron nanoparticles 100 mg/L (28 compounds with a relative percentage of 99.99%). Similarly, in the study of [47] on *M. officinalis*, the highest number of compounds belonged to the essential oil of the control sample (37 compounds) and the lowest number of compounds was related to the essential oil of the sample treated with citrate chelate (17 compounds) [48] reported 35 compounds (85–97%) affected by different levels of jasmonic and salicylic acid treatments for the essential oil of this species, which is contrary to the present results. Today, nanoparticles are widely used to increase crop growth and production, improve germination, produce bioactive compounds, create genetic changes, and protect plants [49].Silver nanoparticles are one of the most widely used nano materials that are used in plant culture to induce growth, eliminate microbial contamination and as an elicitor [50]. Studies have shown that silver nanoparticles have been used to increase the production of secondary metabolites [51].

According to the results, the highest amount of monoterpene compounds in the essential oil of *M. officinalis* leaves, such as monoterpenes hydrocarbons and oxygenated monoterpenes, was observed in (40 mg/L; 22.41%) and (control; 68.04%) nanosilver spray treatments; respectively. The highest amount of oxygenated monoterpenes for the essential oil of *M. officinalis* from the Algiers region with a value of 84.98% was reported by [27]. Oxygenated monoterpenes by [46] in Serbia (0.73–0.80 percent) and by [52] with a value of 70.72% in Germany were the dominant group of compounds of this essential oil. Also, the sesquiterpene compounds of the essential oil, including Sesquiterpenes hydrocarbons and Oxygenated sesquiterpenes, were the main compounds in the samples sprayed with nano iron treatment of 60 mg/L with the amount of 40.75% and 32.72%, respectively. Similarly, [48] reported an increase in the group of oxygenated compounds and sesquiterpene hydrocarbons for *M. officinalis* essential oil under jasmonic and salicylic acid foliar treatments. Environmental factors cause changes in the growth of medicinal plants as well as the quantity and quality of essential oils. The place of growth and development of medicinal plants, in terms of topography and edaphics (elevation and soil characteristics) are very important in the metabolism of medicinal plants and the changes and synthesis of their effective substances. And it affects how the quantity of the effective substance of medicinal and aromatic plants changes [4].

**Fig 1:**
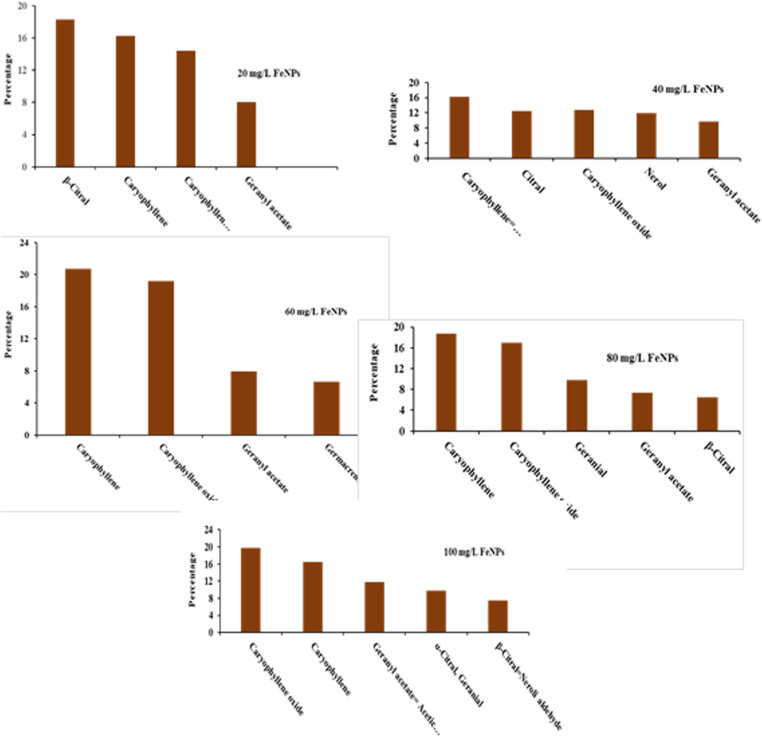
Dominant compounds of foliar treatment with iron nanoparticles.

**Fig 2:**
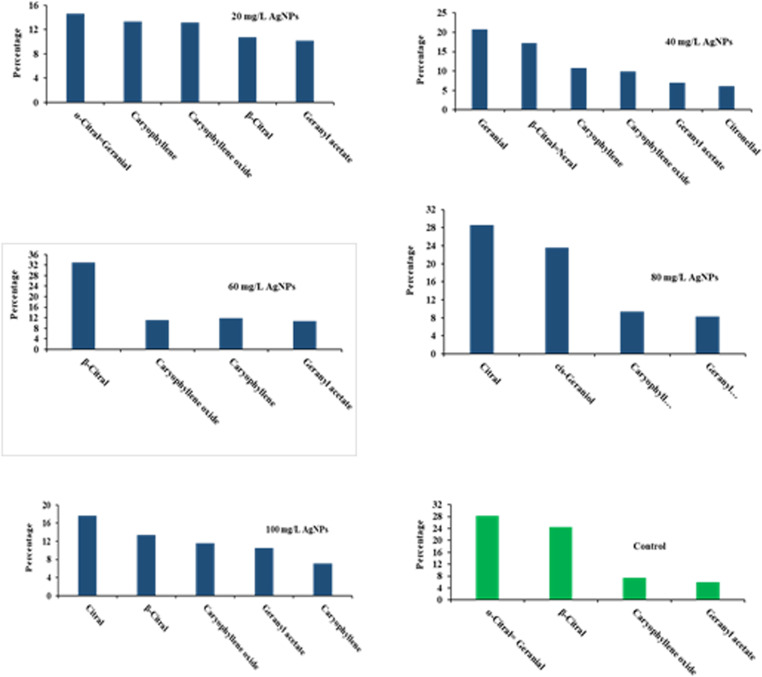
Dominant compounds of foliar treatment with silver nanoparticles and control sample.

The results of the analysis of variance showed that foliar spraying with different treatments of nano iron and silver had a significant effect on the percentage of essential oil compounds of *M. officinalis* (p ≤ 0.01) (Figs 1 and 2). Based on the results obtained, among the constituents of *M. officinalis* leaf essential oil, β-citral or neral (0–33.5%), citral (0–28.53%), geranial (0–28.25%), caryophyllene (0–20.71%), caryophyllene-oxide (7.36–73.19%) and geranial acetate (5.99–11.84%) were the dominant and main compounds in different treatments. Similarly, [48] for *M. officinalis* essential oil under the foliar treatments of jasmonic and salicylic acid respectively geranial (48.33%), neral (33.65%), carvacrol (14.76%), citronellal (46%) reported thymol (4.37%), and caryophyllene (5.3%) as main compounds [53] found the main compounds of the essential oil of *M. officinalis* under the influence of foliar spraying of iron and zinc nutrients in Khorramabad, respectively, geranial (66.65%), trans-caryophyllene (8.41%), caryophyllene oxide (4.99%), verbanol (2.17%), citronellol (1.6 percent), α-morolol (1.52%), germacrine-D (1.38%) and α- cadinol (1.33%). Studies have shown that there is a strong relationship between the pathway of primary metabolism and the biosynthesis of secondary metabolism, and the proper correlation of carbon assimilation and the accumulation of secondary metabolites is dependent on several internal and external factors, especially the optimal levels of micronutrient elements [54]. The existence of some differences in our results with previous studies, such as a decrease in the amount of different compounds and the type of essential oil compounds, can be attributed to ecological changes such as the climate and soil of the cultivation area [53].

Based on the results of *M. officinalis* leaf essential oil in the foliar treatment with nano silver 60 mg/L, the dominant compound of β-citral (neral) was 33.05%, which was the highest amount compared to all treatments and the control (S12 Fig in S1 File). Similarly, [55] reported the highest amount of neral compound under cow dung treatment with a value of 7.6 for this essential oil.

According to the results of *M. officinalis* leaf essential oil, the highest amount of citral compound (28.53%) was observed in the sample treated with nano silver 80 mg/L compared to other treatments and the control (S13 Fig in S1 File). Similarly, this compound has been registered by [56] with a value of 14.2% in the second position of *M. officinalis* essential oil compounds. This compound was not present in other treatments, except for the samples treated with foliar spray with 100 mg/L of silver (17.65%) and 40 mg/L of iron (12.47%). Citral is used in the preparation of bakery and ice cream products, cosmetics, perfume and cologne, candy making, and is also used as a flavoring agent [57].

The findings showed that the highest amount of geranial in the essential oil of *M. officinalis* leaves was 28.25% in the control treatment (S14 Fig in S1 File). Similarly, this compound with the amount of 7.78% from Serbia [46], 66.65% from Khorramabad [53], 41.33% from the foliar treatment with 0.5 mg per A liter of jasmonic acid [48] and 45.6% from the Algiers region [27] have been recorded as the first composition of this essential oil. On the other hand, the results showed that the composition of geranial was not observed at all in the samples sprayed with nano iron 20, 40 and 60 mg/l and nano silver 60, 80 and 100 mg/L treatments [17] reported for *Cichorium intybus* L. essential oil that zinc and iron sulfate foliar spraying had no effect on the amount of kaempferol compound, which is consistent with the present results. The absence of the geranial compound has been reported in the Nafza and Tabarka regions of Tunisia [52]. The lowest amount of this compound was recorded from Dumasnea region of Romania, 2.8% [58]. Geranial is found in the vegetative tissues of many medicinal plants and in some cases it is the main ingredient of essential oils and is naturally present in many essential oils. This compound is insoluble in water and soluble in alcohol and ether, and it is also found in ester or free form. Also, this combination is used in the soap making, cosmetic and perfume industries [17].

The fourth dominant compound of *M. officinalis* leaf essential oil was caryophyllene or β-caryophyllene, the highest amount of which (20.71%) was observed in the nano iron treatment of 60 mg/liter compared to other treatments and the control sample (S15 Fig in S1 File). Similarly, [55] found the highest amount of caryophyllene under the influence of sheep and chicken manure treatments with an amount of 6.4–5.8%, [48] 5.3% and [59] recorded the highest amount of caryophyllene under the effect of foliar spraying treatment of 200 mg/L chitosan in the flowering stage (6.84%) for this essential oil. On the other hand, the results showed that caryophyllene compound was not observed in the sample without foliar application (control). The lowest amount of this compound in the control treatment of Khorramabad region was 0.89% by [53]. On the other hand, the results showed that the amount of caryophyllene in the treatments of 80 and 100 mg/L of nano-iron had a decreasing trend, which is in line with the results of [53] regarding the composition of caryophyllene oxide in the same essential oil. It seems that this is caused by the toxicity of the iron element on this compound. In the conditions of iron toxicity (high amounts), the lack of neutralization of oxygen radicals and the remaining hydrogen peroxide in the plant leads to the Fenton and Haber-Weiss reaction, during which a dangerous hydroxide radical is produced, which can successively destabilize various biological macromolecules, including lipids and proteins. And after that, the amount of production of compounds such as caryophyllene oxide will decrease. The result of oxidative stress caused by iron toxicity in plants is the reduction of proteins, soluble sugars, chlorophyll and irreversible damage to biological membranes and nucleic acids, which has been reported by many researchers [60,61]. Caryophyllene is one of the main compounds of *M. officinalis* with the formula of a bicyclic sesquiterpene. It is used as a flavoring agent for gum, chewing gum and spices, and its commercial use is as a light yellow colored liquid. There are buds in different parts of the plant such as leaves and stems and it is obtained from many plants such as cloves [62]. Caryophyllene has an anticancer effect and has the ability to prevent Alzheimer’s, MS, diabetes, cardiovascular and neurological adverse effects [63].

The fifth dominant compound of *M. officinalis* leaf essential oil was caryophyllene oxide, the highest amount of which was observed in the foliar-sprayed samples with 100 mg/L nano-iron foliar treatment (19.73%) (S16 Fig in S1 File). According to the results, among all the treatments applied to *M. officinalis*, caryophyllene oxide was the lowest in the control treatment with 7.36%. Similarly, [47] recorded the lowest amount of caryophyllene oxide in the control treatment with a value of 2.37%. The lowest amount of caryophyllene oxide was reported by [27] with a value of 0.310%. Caryophyllene oxide is anti-tumor and prevents abnormal fluid accumulation in the intercellular membrane of body tissues [64]. The use of micronutrients in the right amount and in the form of foliar spraying is effective in the growth and production of effective substances of plants and leads to an increase in the percentage of compounds that make up the medicinal plant essential oil and improves the condition of the plant [38]. According to the study of [55] some compounds in the essential oil of *Thymus vulgaris* L., including thymol and carvacrol, were increased by foliar application of iron and zinc micronutrients. Also, in some plants, there is a close relationship between photosynthesis, the production of terpenoids, and photorespiration, and the increase in essential oil compounds such as caryophyllene oxide can be attributed to the role of iron in increasing photosynthesis. Studies have shown that the path of primary metabolism and secondary biosynthesis have a strong relationship, and the composition of secondary metabolites is dependent on several external and internal factors, especially the optimal levels of micronutrient elements [53,54].

According to the results, among all applied foliar treatments, the sixth dominant compound of *M. officinalis* leaf essential oil was geranial acetate, the highest amount of which was observed in the samples treated with 100 mg/L nano-iron foliar spray at an amount of 11.84% (S17 Fig in S1 File). The highest amount of this compound was under treatments of horse manure with the amount of 17.7% by [65] and under the treatments of spraying jasmonic acid (0.14 g/L) and salicylic acid (0.40 g/L) in both with an amount of 5 2.0% was recorded for this essential oil. This is while in the present study, this compound was in the third place in the essential oil of the samples treated with foliar spraying with nano iron 100 mg/liter [56] reported the amount of geranyl acetate as 27.9% and as the first dominant compound of *M. officinalis* essential oil. Also, geranyl acetate was reported by [45] with the amount of 1.90% and as the fifth compound, and by [52] with the amount of 1.42% in the German region and as the seventh compound. The lowest amount of this compound in the present study was observed in the control treatment with a value of 5.99%. The lowest amount of geranial acetate was recorded by [27] with 0.5% from Algeria.

### 3.3 Antimicrobial activity

The results of investigating the antimicrobial activity of the essential oil of *M. officinalis* under different foliar treatments with different levels of iron and silver nanoparticles by agar diffusion method against different bacterial strains are shown in Table 2. The results of the analysis of variance showed that there was a significant difference between the diameter of the growth inhibition zone created by *M. officinalis* essential oil under different treatments and control antibiotics against different bacterial strains. (p ≤ 0.01) (Table 2). The essential oil of *M. officinalis* leaves did not inhibit the growth of any of the studied bacterial strains under different treatments. Similarly, [27] reported the absence of a growth inhibition zone against Gram-positive bacteria *S. aureus* and [45] against *P. aeruginos* bacteria by *M. officinalis* essential oil. Considering that the antimicrobial effect of plant essential oils is not limited to one mechanism and only the effect on the cell wall, perhaps the reason for this similarity can be justified in terms of lack of inhibitory activity [66]. According to previous studies, the diameter of the growth inhibition zone of *M. officinalis* essential oil against the gram-positive bacteria *S.aureus* by [67] equals 20.8 mm, by [68] was equal to 15.33 mm and by [45] it was 13 mm. Also, based on previous studies, the diameter of the growth inhibition zone of *M. officinalis* essential oil against Gram-positive bacteria *S. epidermidis* was recorded as 18 mm by [45] and 16 mm by [67]. The only report of growth inhibition zone by *M. officinalis* essential oil against *P. aeruginos* was equal to 50 mm [27]. It seems that the variability and diversity in the inhibitory effects of essential oils on different microbes is due to the difference in different strains of the same microbial species and the difference in the main compounds that make up the essential oil in different chemotypes of the same species in different environmental conditions [69,70].

**Table 2:** MIC and MBC values of different essential oils treated with silver and iron nanoparticles, and control antibiotics against bacterial strains.

	Traeatment	*Staphylococcus epidermidis*	*Staphylococcus aureus*	*Pseudomonas aeruginos*
MIC (μg/mL)	Fe-20	2000	1000	1000
	Fe-40	500	500	500
	Fe-60	2000	2000	500
	Fe-80	1000	1000	500
	Fe −100	500	125	<62.5
	Ag −20	1000	500	<62.5
	Ag −40	500	500	500
	Ag-60	125	125	<62.5
	Ag-80	1000	500	500
	Ag −100	1000	500	500
	Control	500	500	500
	Rifampin	1.95	3.9	31.25
	Gentamicin	3.9	7.8	7.8
MBC (μg/mL)	FE-20	2000	1000	2000
	Fe-40	500	500	2000
	Fe-60	1000	1000	1000
	Fe-80	1000	1000	2000
	Ag-100	500	250	1000
	Ag −20	2000	500	2000
	Ag −40	500	500	2000
	Ag-60	250	500	1000
	Ag-80	1000	500	2000
	Ag −100	1000	500	2000
	Control	500	500	2000
	Rifampin	7.8	3.9	250
	Gentamicin	3.9	7.8	15.63

The results of analysis of variance showed that there was a significant difference between the MIC value of *M. officinalis* leaf essential oil under the different treatments studied and control antibiotics against different bacterial strains. (p ≤ 0.01) (Table 2). The lowest MIC value belonging to the treatments of nano iron 100, nano silver 20, and nano silver 60 mg/L was observed with a value of <62.5 μg/mL against *P. aeruginos*, which is one times weaker than rifampin (MIC = 31.25 μg/mL), and three times weaker than gentamicin (MIC = 7.8 μg/mL). This is while compared to the essential oil of *M. officinalis*, the control sample (MIC = 500 µg/ml) was three times stronger against this bacterium.

The difference in antimicrobial effects indicates the differences in the composition of essential oils [71]. Studies have shown that among Gram-negatives, *Pseudomonas* has the least sensitivity to the antimicrobial effect of essential oils [72], which is contrary to the present results. Plant essential oils can prevent the growth of microorganisms through various mechanisms such as affecting the cell wall, preventing the production of proteins, preventing the functioning of the cytoplasmic membrane, etc. In most of the research, the effect on the cell wall of bacteria and the difference in the type of cell wall of Gram-positive and Gram-negative bacteria have been mentioned as the main reason for the greater antimicrobial effect of essential oils [73]. Also, previous studies show that Gram-negative bacteria are less sensitive to the antibacterial effects of essential oils. Perhaps the reason is the existence of an outer membrane in the cell wall of Gram-negative bacteria, which limits the diffusion of the hydrophobic components of the essential oil into the bacterial cell. Of course, exceptions have been observed that Gram-positive sensitivity is not higher and shows a high sensitivity to essential oils. Different degrees of activity against Gram-negative or positive bacteria are observed in each component of the essential oil [72,74].

Another significant inhibitory activity of *M. officinalis* leaf essential oil against Gram-positive *S.aureus* with an MIC value of 125 μg/mL was obtained by the essential oil sample treated with nano iron 100 mg/L and nano silver 60 mg/L which compared to rifampin antibiotics (MIC = 3.9 μg/mL) 5 times and gentamicin (MIC = 7.8 μg/mL) worked 4 times weaker. This is while comparing the essential oil sample of the control treatment (MIC = 500 µg/ml) it had a stronger effect against this bacterium. Similarly, the MIC value of *M. officinalis* essential oil against *S.aureus* was reported by [45,72]. has been It seems that the presence of the dominant compounds caryophyllene, caryophyllene oxide, geranyl acetate and neral on the one hand and the presence of the minor compound thymol in the essential oil sample under the treatments of nano iron 100 mg/liter and nano silver 60 mg/liter are possible factors of this effect. Antimicrobial substances and phenolic compounds such as thymol in Gram-positive bacteria such as *Staphylococcus aureus* by destroying the cell wall and its membrane, cause the leakage of substances inside the bacteria to the outside [75]. Also, according to the studies, phenolic compounds exert their antimicrobial activity by increasing the permeability of the cell membrane, its swelling and ultimately cell death [76]. The antibacterial effect of caryophyllene has been confirmed against Gram-positive bacteria *S. aureus* [77].

On the other hand, the results showed that the lowest MIC value of the essential oil of *M. officinalis* against Gram-positive *S. epidermidis* (125 μg/mL) was observed in the essential oil sample treated with nano silver 60 mg/L, which has worked six times and 5 times weaker rather than to rifampin antibiotics (MIC = 1.95 μg/mL) and gentamicin (MIC = 3.9 μg/mL); respectively. Similarly, [45] reported the MIC value of *M. officinalis* essential oil against S. epidermidis as 31.25 μg/mL, which is stronger than our results. This is while the value of the essential oil sample of the control treatment was equal to 500 µg/mL. It seems that the predominance of neral compound in the essential oil treated with 60 mg/L nano silver compared to other samples is one of the possible factors of this antibacterial activity. Also, the presence of minor compounds such as carvacrol, damascenone, and 1, 2, 4, 5 tetramethyl benzene only in the essential oil treated with nano silver 60 mg/L can be other possible factors affecting this antibacterial activity. Carvacrol is a phenolic monoterpene and a colorless and slightly viscous liquid that darkens in the presence of air. It is widely used as a disinfectant, production of health products, insect repellants, deodorizing sprays. Carvacrol is also used in the production process of synthetic essential oils [78]. carvacrol has antimicrobial, insecticidal and antipyretic properties, and no toxic effects have been reported [79,80]. In fact, carvacrol is an isomer of thymol and has a smell similar to thymol (White, 2011). On the other hand, the presence of thymol compound in this essential oil along with carvacrol can be another possible effective factor. Carvacrol and thymol have different biological and medicinal properties such as antioxidant, antibacterial, antifungal, anticancer, anti-inflammatory, hepatosteroid, spasmolytic and vasorelaxant [81]. The antimicrobial effect of carvacrol and thymol is due to the permeability of the cell membrane by these substances, which can bind to the cations on the surface of the pathogen’s membrane and disrupt their vital activities. It has antimicrobial effects through the reaction with the membrane of microorganisms and changes in the permeability of compounds such as potassium and hydrogen [82,83].

The sensitivity of Gram-positive bacteria is related to the direct interaction of the hydrophobic components of essential oils with the cell wall [84]. Gram-positive bacteria have a wall with a thick layer of peptidoglycans (90–95%), proteins and nucleic acid. The main parts of the essential oil have a hydrophobic nature and can easily pass through it. On the other hand, in Gram-negative bacteria, the monolayer wall of peptidoglycans is surrounded by an outer layer of lipopolysaccharides and proteins and is more complex. This cell’s outer membrane is charged with a hydrophilic nature and limits the diffusion of hydrophobic compounds through the LSP [85]. Therefore, Gram-positive bacteria can be easily inhibited by essential oils compared to Gram-negative bacteria due to structural changes in the outer layer of bacteria [86]. The compounds of essential oils mainly affect the membrane of cells and depending on their hydrophobicity and the cell wall, the permeability of the cell membrane of microorganisms to the compounds of essential oils is determined. Therefore, the difference in the cell membrane structure of Gram-positive or Gram-negative bacteria causes microorganisms to have different responses to essential oils and their effective compounds [87–91].

## 4 Conclusion

*M. officinalis* is one of the important medicinal plants, and the cultivation of this plant is of great interest due to the compounds in its essential oil. Since agricultural ecosystems and especially the management of nutritional elements have a determining role in the biosynthesis of secondary metabolites of medicinal plants, the present study was designed and implemented with the aim of foliar application of iron and silver nanoparticles on the quantity, quality, and antimicrobial activity of *M. officinalis* leaf essential oil. became. The quantitative analysis of the essential oil showed that the best treatment in terms of efficiency was observed at the samples sprayed with iron nanoparticles 60 mg/L (1.70%), which is 20 times more than the efficiency of the control sample, and it was often higher than all the reported yields of this essential oil in previous studies. Qualitative analysis and chemical profile of the studied essential oils, showed the predominance of neral (33.05%) in the foliar treatment with nano silver 60 mg/L, citral (28.53%) in the sample treated with nano silver foliar treatment 80 mg/L, geranial (28.25%) in the control treatment, caryophyllene (20.71%) in the nano iron treatment 60 mg/L, caryophyllene oxide (19.73%) in the foliar treatment with nano iron 100 mg/L and geranyl acetate (11.84%) in the foliar treatment with iron nanoparticles 100 mg/L. In erms of antibacterial property, the samples sprayed with 60 mg/L nano silver treatment were effective against *P. aeruginos*, *S. aureus* and *S. epidermidis*, which was probably caused by the chemical profile and especially the unique appearance of phenolic compounds such as carvacrol. Therefore, it can be concluded that by using appropriate amounts of nano-fertilizers, in addition to reducing fertilizer consumption and environmental pollution, it is possible to move towards sustainable agriculture by improving the quantitative and qualitative characteristics of *M. officinalis*. This study may encourage farmers to find the optimal concentrations of iron and silver nanoparticles for different medicinal and aromatic plants for better quality and increased production of essential oils. In addition, this technique is cost-effective because nanoparticles are only effective at low concentrations.

## Supporting information

S1 File The Supporting Information file contains S1–S17 Figs and S1–S11 Tables. (DOCX)
