# Supplementary material for: The effect of foliar spraying of silver and iron nanoparticles as fertilizers on the quantity, quality, and antimicrobial properties of Melissa officinalis L. essential oil
Source: PLoS One. 2025 Jun 5;20(6):e0323296. doi: 10.1371/journal.pone.0323296 (PMC12140251; doi:10.1371/journal.pone.0323296)
Supplement: S1 File — (DOCX) [file pone.0323296.s001.docx]

**Supplementary materials**

**Table S1- Chemical composition of *M. Officinalis* leaf essential oil under the influence of iron nanoparticle foliar spraying of 20 mg/L**

| **2** | **Compound** | **RI^*^** | **RI** | **Mean (%) ± SD** | **Molecular formula** |
| --- | --- | --- | --- | --- | --- |
| 1 | Citronellal | 1153.4 | 1170 | 1.15 | C_10_H_18_O |
| 2 | 3, 6-Octadienal3,7 -dimethyl- | 1181.1 | 1183.9 | 1.86 | C_10_H_16_O |
| 3 | Citronellol | 1230.1 | 1217 | 1.42 | C_10_H_20_O |
| 4 | β-Citral; cis-Citral; Neral | 1273.4 | 1235.0 | 18.29 | C_10_H_16_O |
| 5 | trans-Geranic acid methyl ester= Geranic acid methyl ester; Methyl geranoate | 1322.2 | 1321.7 | 1.71 | C_11_H_18_O_2_ |
| 6 | Geranyl acetate | 1379.5 | 1383 | 8.03 | C_12_H_20_O_2_ |
| 7 | α-Cubebene | 1383.2 | 1351 | 2.2 | C_15_H_24_ |
| 8 | β-Bourbonene | 1392.2 | 1384 | 3.31 | C_15_H_24_ |
| 9 | Cyclohexane=beta elemene | 1395.3 | 1391 | 1.24 | C_15_H_24_ |
| 10 | Benzene, 1,2,3,5-tetramethyl- =Isodurene | 4095.1 | 1115 | .0.78 | C_10_H_14_ |
| 11 | Caryophyllene | 1418.1 | 1451 | 16.25 | C_15_H_24_ |
| 12 | β-Cubebene | 1426.2 | 1390 | 0.48 | C_15_H_24_ |
| 13 | trans-Geranylacetone= Geranylaceton | 1438.7 | 1454 | 0.44 | C_13_H_22_O |
| 14 | Humulene =α-Caryophyllene, | 1458.6 | 1488 | 2.14 | C_15_H_24_ |
| 15 | epi-β-Caryophyllene | 1463.5 | 1467 | 0.55 | C_15_H_24_ |
| 16 | Germacrene D | 1488.9. | 1485 | 5.31 | C_15_H_24_ |
| 17 | β-Gurjunene (calarene) | 5005.1 | 1409 | 0.54 | C_15_H_24_ |
| 18 | Cadina-1(10),4-diene | 5025 | 1562 | 1.38 | C_15_H_24_ |
| 19 | Espatulenol; Spatulenol | 5090.4 | 1622 | 0.8 | C_15_H_24_O |
| 20 | Caryophyllene oxide= β-Caryophyllene epoxide | 5096.5 | 1583 | 14.4 | C_15_H_24_O |
| 21 | Humulene 6,7-epoxide | 6175.3 | 1607 | 0.66 | C_15_H_24_O |
| 22 | 11,11-Dimethyl-4,8-dimethylenebicyclo[7.2.0]undecan-3-ol = | 1643.3 | 1645.9 | 1.52 | C_15_H_24_O |
| 23 | α-Cadinol | 1667.6 | 1653 | 2.59 | C_15_H_26_O |
| 24 | Caryophyllenol-II | 1687.8 | 1675 | 1.58 | C_15_H_24_O |
| 25 | Heptadecane= n-Heptadecane | 7000.6 | - | 1.15 | C_17_H_36_ |
| 26 | Perhydrofarnesyl acetone=Hexahydrofarnesyl acetone | 8041.9 | 1855 | 0.88 | C_18_H_36_O |
| 27 | 1-Nonadecene | 8074.9 | 1894 | 1.56 | C_19_H_38_ |
| 28 | Nonadecane | 1704.9 | - | 4.97 | C_19_H_40_ |
| 29 | n-Hexadecanoic acid=Hexadecanoic acid=Hydrofol | 2094.2 | 1972 | 0.8 | C_16_H_32_O_2_ |
| 30 | Eicosane | 2100 | - | 0.48 | C_20_H_42_ |
| 31 | Heneicosane | 2053 | - | 1.55 | C_21_H_44_ |
|  | Total |  |  | 100 |  |
|  | Monoterpenes hydrocarbons |  |  | 0.78 |  |
|  | Oxygenated monoterpenes |  |  | 22.72 |  |
|  | Sesquiterpenes hydrocarbons |  |  | 33.4 |  |
|  | Oxygenated sesquiterpenes |  |  | 21.55 |  |
|  | Others (Nonterpenoids) |  |  | 21.57 |  |

RI refers to the retention index identified by database NIST 014; RI*refers to the retention index calculated from the retention time relative to that of C8 – C40 n-alkanes

**Table S2- Chemical composition of *M. Officinalis* leaf essential oil under the influence of 40 mg/L iron nanoparticle foliar spraying**

| **no.** | **Compound** | **RI^*^** | **RI** | **Mean (%) ± SD** | **Molecular formula** |
| --- | --- | --- | --- | --- | --- |
| 1 | Linalool | 1102 | 1080 | 0.46 | C_10_H_18_O |
| 2 | Citronellal | 1153 | 1170 | 1.56 | C_10_H_18_O |
| 3 | 3,6-Octadienal, 3,7-dimethyl- | 1181 | 1183.9 | 2.12 | C_10_H_16_O |
| 4 | Benzenamine, 3-methoxy-=m-Anisidine | 1212 | _ | 0.48 | C_7_H_9_NO |
| 5 | Nerol, cis-Geraniol | 1244 | 1235 | 11.87 | C_10_H_16_O |
| 6 | Geraniol,Lemonol | 1150 | 1252 | 0.64 | C_10_H_18_O |
| 7 | 6-Octenoic acid, 3,7-dimethyl-, methyl ester= Methyl citronellate=Citronellic acid | 1257 | 1260 | 0.63 | C_11_H_20_O_2_ |
| 8 | 2,6-Octadienal, 3,7-dimethyl-, (Z)-= β-Citral, | 1275 | _ | 12.47 | C_10_H_16_O |
| 9 | Thymol | 1301 | 1294 | 0.53 | C_10_H_14_O |
| 10 | Carvacrol, Antioxine | 1310 | 1294 | 0.52 | C_10_H_14_O |
| 11 | trans-Geranic acid methyl ester=Geranic acid methyl ester, Methyl geranoate | 1323 | 1321.7 | 2.49 | C_11_H_18_O_2_ |
| 12 | 2,6-Octadien-1-ol, 3,7-dimethyl-, acetate, (Z)-= Nerol acetate | 1358 | 1363 | 0.51 | C_12_H_20_O_2_ |
| 13 | Geranyl acetate; Acetic acid, geraniol ester, Geraniol acetate, 2,6-Octadien-1-ol, 3,7-dimethyl-, acetate, (E)- | 1380 | 1383 | 9.64 | C_12_H_20_O_2_ |
| 14 | α-Cubebene | 1383 | 1354 | 1.99 | C_15_H_24_ |
| 15 | (-)-β-Bourbonene | 1392 | 1384 | 3.4 | C_15_H_24_ |
| 16 | Cyclohexane, 1-ethenyl-1-methyl-2,4-bis(1-methylethenyl)-, [1S-(1α,2β,4β)]- = β-Elemene, | 1395 | 1391 | 0.73 | C_15_H_24_ |
| 17 | p-Mentha-1,5,8-triene= Scifinder, | 4099 | 1111 | 1.39 | C_10_H_14_ |
| 18 | Caryophyllene= β-Caryophillene, | 1419 | 1451 | 16.22 | C_15_H_24_ |
| 19 | β-Copaene | 1426 | 1428 | 0.47 | C_15_H_24_ |
| 20 | trans-Geranylacetone, Acetone, geranyl- ; Geranyl acetone | 1439 | 1454 | 0.37 | C_13_H_22_O |
| 21 | cis-β-Farnesene | 1444 | 1428 | 0.75 | C_15_H_24_ |
| 22 | 2,6,10-Trimethyltridecane= Tridecane, 2,6,10-trimethyl- | 1453 | 1461 | 0.58 | C_16_H_34_ |
| 23 | Humulene | 1458 | 1488 | 1.77 | C_15_H_24_ |
| 24 | Alloaromadendrene | 1463 | 1458 | 0.77 | C_15_H_24_ |
| 25 | Germacrene D | 1489 | 1519 | 4.55 | C_15_H_24_ |
| 26 | Pentadecane | 5000 | _ | 0.41 | C_15_H_24_ |
| 27 | β-Gurjunene | 5005 | 1434 | 0.66 | C_15_H_24_ |
| 28 | δ-Cadinene | 5025 | 1524 | 1.04 | C_15_H_24_ |
| 29 | Caryophyllene oxide | 5062 | 1589 | 12.69 | C_15_H_24_O |
| 30 | Espatulenol; Spatulenol | 5097 | 1622 | 1.00 | C_15_H_24_O |
| 31 | Hexadecane= Cetane | 5099 | - | 0.46 | C_16_H_34_ |
| 32 | Humulene-1,2-epoxide | 1611 | 1670 | 0.59 | C_15_H_24_O |
| 33 | 10,10-Dimethyl-2,6-dimethylenebicyclo[7.2.0]undecan-5β-ol | 1643 | 1644.2 | 1.53 | C_15_H_24_O |
| 34 | α-Cadinol= (E)-α-Cadinol | 1662 | 1660 | 1.54 | C_15_H_26_O |
| 35 | Caryophyllenol-II | 1680 | 1676 | 1.39 | C_15_H_24_O |
| 36 | 1-Cyclohexene-1-carboxaldehyde, 4-(1-methylethyl)-=Phellandral | 1696 | 1273 | 0.42 | C_10_H_16_O |
| 37 | 2-Pentadecanone, 6,10,14-trimethyl- | 8042 | 1855 | 1.18 | C_18_H_36_O |
| 38 | 1,2-Benzenedicarboxylic acid, bis(2-methylpropyl) ester=Phthalic acid, diisobutyl ester, 1,2-Benzenedicarboxylic acid, di(2-methylpropyl) ester | 8061 | 1869 | 0.65 | C_16_H_22_O_4_ |
| 39 | n-Hexadecanoic acid= Hexadecanoic acid; ,Hexadecylic acid; Hydrofol | 2093 | 1964 | 0.46 | C_16_H_32_O_2_ |
|  | Total |  |  | 99.55 |  |
|  | Monoterpenes hydrocarbons |  |  | 1.39 |  |
|  | Oxygenated monoterpenes |  |  | 29.2 |  |
|  | Sesquiterpenes hydrocarbons |  |  | 32.76 |  |
|  | Oxygenated sesquiterpenes |  |  | 19.21 |  |
|  | Others (Nonterpenoids) |  |  | 16.99 |  |

RI refers to the retention index identified by database NIST 014; RI*refers to the retention index calculated from the retention time relative to that of C8 – C40 n-alkanes

**Table S3- Chemical composition of *M. Officinalis* leaf essential oil under the influence of iron nanoparticle foliar spraying of 60 mg/L**

| **no.** | **Compound** | **RI^*^** | **RI** | **Mean (%) ± SD** | **Molecular formula** |
| --- | --- | --- | --- | --- | --- |
| 1 | 3-Methyl-2-(2-methyl-2-butenyl)-furan=Rosefuran | 1192 | 1104.3 | 0.37 | C_10_H_14_O |
| 2 | Citronellal= β-Citronellal; Rhodinal=Citronellel | 1153 | 1154 | 1.25 | C_10_H_18_O |
| 3 | 3,6-Octadienal, 3,7-dimethyl- | 1181 | 1183.9 | 0.49 | C_10_H_16_O |
| 4 | Citronellol= β-Citronellol,Rodinol | 1229 | 1232 | 0.91 | C_10_H_20_O |
| 5 | 2,6-Octadienal, 3,7-dimethyl-, (Z)-= β-Citral | 1242 | 1235 | 3.04 | C_10_H_16_O |
| 6 | 6-Octenoic acid, 3,7-dimethyl-, methyl ester= Methyl citronellate,Citronellic acid, methyl ester | 1257 | 1260 | 0.62 | C_11_H_20_O_2_ |
| 7 | 2,6-Octadienal, 3,7-dimethyl-, (E)- | 1271 | 1269 | 4.85 | C_10_H_16_O |
| 8 | trans-Geranic acid methyl ester; E-Methylgeranate, | 1322 | 1321 | 1.67 | C_11_H_18_O_2_ |
| 9 | Geranyl acetate= 2,6-Octadien-1-ol, 3,7-dimethyl-, acetate, (E)-; | 1379 | 1383 | 7.96 | C_12_H_20_O_2_ |
| 10 | α-Cubebene | 1383 | 1354 | 1.88 | C_15_H_24_ |
| 11 | (-)-β-Bourbonene | 1392 | 1384 | 3.01 | C_15_H_24_ |
| 12 | β-Elemen | 1395 | 1391 | 1.19 | C_15_H_24_ |
| 13 | Benzene, 1-ethyl-2,3-dimethyl | 4096 | 1106 | 0.82 | C_10_H_14_ |
| 14 | Caryophyllene | 1418 | 1451 | 20.71 | C_15_H_24_ |
| 15 | β-Copaene | 1426 | 1433 | 0.52 | C_15_H_24_ |
| 16 | cis-β-Farnesene | 1444 | 1476 | 0.61 | C_15_H_24_ |
| 17 | Humulene | 1458 | 1456 | 2.54 | C_15_H_24_ |
| 18 | Alloaromadendrene | 1463 | 1458 | 0.58 | C_15_H_24_ |
| 19 | Germacrene D | 1489 | 1458 | 6.68 | C_15_H_24_ |
| 20 | α-Muurolene | 5004 | _ | 0.7 | C_15_H_24_ |
| 21 | δ-Cadinene | 5025 | 1568 | 1.63 | C_15_H_24_ |
| 22 | Caryophyllene oxide | 5062 | 1589 | 19.2 | C_15_H_24_O |
| 23 | Germacrene D-4-ol; | 5090 | 1574 | 1.67 | C_15_H_26_O |
| 24 | Ketone, 3aα,4,5,6,7,7aβ-hexahydro-7β-isopropyl-4-methylene-1β-indanyl methyl (8CI) | 1601 | 1611 | 0.47 | C_15_H_24_O |
| 25 | Humulene-1,2-epoxide | 1611 | 1606 | 0.86 | C_15_H_24_O |
| 26 | 10,10-Dimethyl-2,6-dimethylenebicyclo[7.2.0]undecan-5β-ol= 10,10-Dimethyl-2,6-dimethylenebicyclo[7.2.0]undecan-5-ol | 1643 | 1644.2 | 2.47 | C_15_H_24_O |
| 27 | Spirojatamol | 1648 | 1592.1 | 0.62 | C_15_H_26_O |
| 28 | α-Cadinol | 1662 | 1660 | 2.91 | C_15_H_26_O |
| 29 | ----- | 1668 |  | 0.72 | _ |
| 30 | Caryophyllenol-II | 1680 | 1675 | 2.13 | C_15_H_24_O |
| 31 | Ethyl tridecanoate | 1691 | 1687 | 0.45 | C_15_H_30_O_2_ |
| 32 | LEDENE OXIDE-(1) | 1699 | _ | 0.79 | C_15_H_24_O |
| 33 | Heptadecane | 7001 | _ | 0.54 | C_17_H_36_ |
| 34 | Naphthalene, 1,2,3,4-tetrahydro-1,6-dimethyl-4-(1-methylethyl)-, (1S-cis)-= Cadina-1,3,5-triene; (-)-Calamenene | 7004 | 1496 | 0.7 | C_15_H_22_ |
| 35 | 2,6,10-Dodecatrienal, 3,7,11-trimethyl-, (E,E)-= E,E-Farnesal, Farnesal | 7056 | 1719 | 0.42 | C_15_H_24_O |
| 36 | 2-Pentadecanone, 6,10,14-trimethyl-= Perhydrofarnesyl acetone | 8041 | 1847 | 0.83 | C_18_H_36_O |
| 37 | Nonadecane | 8070 | _ | 1.33 | C_19_H_40_ |
| 38 | n-Hexadecanoic acid=Hexadecanoic acid, Palmitinic acid; | 2096 | 1964 | 1.4 | C_16_H_32_O_2_ |
| 39 | Heneicosane | 1992 | _ | 0.47 | C_21_H_44_ |
|  | Total |  |  | 100.01 |  |
|  | Monoterpenes hydrocarbons |  |  | 0.82 |  |
|  | Oxygenated monoterpenes |  |  | 10.91 |  |
|  | Sesquiterpenes hydrocarbons |  |  | 40.75 |  |
|  | Oxygenated sesquiterpenes |  |  | 32.72 |  |
|  | Others (Nonterpenoids |  |  | 14.03 |  |

RI refers to the retention index identified by database NIST 014; RI*refers to the retention index calculated from the retention time relative to that of C8 – C40 n-alkanes

**Table S4- Chemical composition of *M. Officinalis* leaf essential oil under the influence of iron nanoparticle foliar spraying of 80 mg/L**

| **no.** | **Compound** | **RI^*^** | **RI** | **Mean (%) ± SD** | **Molecular formula** |
| --- | --- | --- | --- | --- | --- |
| 1 | trans-β-Ocimene= (3E)-3,7-Dimethyl-1,3,6-octatriene | 1047 | 1050 | 0.35 | C_10_H_14_ |
| 2 | Rosefuran | 1092 | 1104.3 | 0.37 | C_10_H_14_O |
| 3 | Linaloo | 1102 | _ | 0.46 | C_10_H_18_O |
| 4 | Citronellal= Rhodinal | 1154 | 1153 | 1.87 | C_10_H_18_O |
| 5 | 3,6-Octadienal, 3,7-dimethyl | 1181 | 1183.9 | 2.04 | C_10_H_16_O |
| 6 | β-Citral | 1245 | 1240 | 6.46 | C_10_H_16_O |
| 7 | 6-Octeoic acid, 3,7-dimethyl-, metyl ester, (S)- | 1257 | _ | 0.77 | C_11_H_20_O_2_ |
| 8 | Geranial | 1275 | 1269 | 9.74 | C_10_H_16_O |
| 9 | trans-Geranic acid methyl ester=Geranic acid methyl ester, Methyl geranoate | 1323 | 1284 | 1.77 | C_11_H_18_O_2_ |
| 10 | Geranyl acetate= Acetic acid, geraniol ester,Geraniol acetate | 1381 | 1379 | 7.37 | C_12_H_20_O_2_ |
| 11 | α-Cubebene | 1383 | 1354 | 2.56 | C_15_H_24_ |
| 12 | (-)-β-Bourbonene | 1393 | 1384 | 3.22 | C_15_H_24_ |
| 13 | β-Elemene | 1396 | 1391 | 1.02 | C_15_H_24_ |
| 14 | Benzene, 1-ethyl-3,5-dimethyl- =; 1-Ethyl-3,5-dimethylbenzene | 1401 | 1059 | 0.86 | C_10_H_14_ |
| 15 | Caryophyllene | 1421 | 1410 | 18.7 | C_15_H_24_ |
| 16 | β-Copaene | 1427 | 1428 | 0.52 | C_15_H_24_ |
| 17 | Geranyl acetone | 1439 | 1454 | 0.45 | C_13_H_22_O |
| 18 | cis-β-Farnesene | 1445 | 1476 | 0.73 | C_15_H_24_ |
| 19 | Humulene= α-Caryophyllene | 1459 | 1453 | 2.43 | C_15_H_24_ |
| 20 | Alloaromadendrene | 1464 | 1453 | 0.63 | C_15_H_24_ |
| 21 | Germacrene D | 1490 | 1480 | 6.03 | C_15_H_24_ |
| 22 | α-Muurolene | 5005 | _ | 0.65 | C_15_H_24_ |
| 23 | Naphthalene, 1,2,3,4,4a,5,6,8a-octahydro-7-methyl-4-methylene-1-(1-methylethyl)-, (1α,4aβ,8aα)- | 5021 | 1513 | 0.35 | C_15_H_24_ |
| 24 | Naphthalene, 1,2,3,5,6,8a-hexahydro-4,7-dimethyl-1-(1-methylethyl)-, (1S-cis)- | 5025 | 1524 | 1.95 | C_15_H_24_ |
| 25 | Caryophyllene oxide | 5099 | 1583 | 17.02 | C_15_H_24_O |
| 26 | Humulene epoxide 2 | 1612 | 1607 | 0.79 | C_15_H_24_O |
| 27 | 10,10-Dimethyl-2,6-dimethylenebicyclo[7.2.0]undecan-5β-ol | 1644 | 1644.2 | 1.58 | C_15_H_24_O |
| 28 | α-Cadinol | 1663 | 1660 | 3.01 | C_15_H_26_O |
| 29 | Caryophyllenol-II | 1681 | 1675 | 1.67 | C_15_H_24_O |
| 30 | 2-Pentadecanone, 6,10,14-trimethyl- | 8042 | 1847 | 0.97 | C_18_H_36_O |
| 31 | Nonadecane | 1700 | - | 1.23 | C_19_H_40_ |
| 32 | n-Hexadecanoic acid= Palmitic acid, Cetylic acid; | 2099 | 1977 | 1.16 | C_16_H_32_O_2_ |
| 33 | Heneicosane | 1927 | - | 1.27 | C_21_H_44_ |
|  | Total |  |  | 100 |  |
|  | Monoterpenes hydrocarbons |  |  | 1.21 |  |
|  | Oxygenated monoterpenes |  |  | 21.71 |  |
|  | Sesquiterpenes hydrocarbons |  |  | 38.79 |  |
|  | Oxygenated sesquiterpenes |  |  | 24.07 |  |
|  | Others (Nonterpenoids |  |  | 14.22 |  |

RI refers to the retention index identified by database NIST 014; RI*refers to the retention index calculated from the retention time relative to that of C8 – C40 n-alkanes

**Table S5- Chemical compounds of *M. Officinalis* leaf essential oil under the influence of 100 mg/L iron nano particle foliar spraying**

| **no.** | **Compound** | **RI^*^** | **RI** | **Mean (%) ± SD** | **Molecular formula** |
| --- | --- | --- | --- | --- | --- |
| 1 | Linalool | 1102 | 1080 | 0.73 | C_10_H_18_O |
| 2 | Citronellal | 1153 | 1154 | 1.15 | C_10_H_18_O |
| 3 | 3,6-Octadienal, 3,7-dimethyl- | 1181 | 1183.9 | 1.08 | C_10_H_16_O |
| 4 | cis-Geraniol, Nerol, (Z)-Geraniol; | 1228 | 1227 | 1.06 | C_10_H_18_O |
| 5 | β-Citral: Neroli aldehyde | 1241 | 1235 | 7.42 | C_10_H_16_O |
| 6 | Methyl citronellate | 1257 | 1242 | 0.52 | C_11_H_20_O_2_ |
| 7 | α-Citral= Geranial | 1271 | 1269 | 9.81 | C_10_H_16_O |
| 8 | trans-Geranic acid methyl ester= Methyl geranoate | 1322 | 1321.7 | 2.27 | C_11_H_18_O_2_ |
| 9 | Nerol acetate; Neryl acetate | 1358 | 1365 | 0.5 | C_12_H_20_O_2_ |
| 10 | Geranyl acetate= Acetic acid, geraniol ester; Geraniol acetate | 1378 | 1379 | 11.84 | C_12_H_20_O_2_ |
| 11 | β-Damascenone | 1383 | 1386 | 2.56 | C_13_H_18_O |
| 12 | (-)-β-Bourbonene | 1391 | 1386 | 2.6 | C_15_H_24_ |
| 13 | Tetradecane | 4002 | _ | 0.63 | C_14_H_30_ |
| 14 | Benzene, 2-ethyl-1,4-dimethyl- | 4092 | 1074 | 2.27 | C_10_H_14_ |
| 15 | Caryophyllene | 1416 | 1420 | 16.47 | C_15_H_24_ |
| 16 | trans-Geranylacetone; Acetone, geranyl-; Geranyl acetone | 1438 | 1454 | 0.51 | C_13_H_22_O |
| 17 | Humulene= α-Caryophyllene | 1458 | 1453 | 1.7 | C_15_H_24_ |
| 18 | Germacrene D | 1487 | 1480 | 3.63 | C_15_H_24_ |
| 19 | Naphthalene, 1,2,3,5,6,8a-hexahydro-4,7-dimethyl-1-(1-methylethyl)-, (1S-cis)- | 5024 | 1524 | 0.94 | C_15_H_24_ |
| 20 | Caryophyllene oxide | 5095 | 1583 | 19.73 | C_15_H_24_O |
| 21 | Hexadecane= Cetane | 6000 | _ | 1.36 | C_16_H_34_ |
| 22 | Humulene 6, 7-epoxide | 1610 | 1607 | 0.79 | C_15_H_24_O |
| 23 | Caryophylla-4(12),8(13)-dien-5.beta.-ol | 1643 | 1644.2 | 2.09 | C_15_H_24_O |
| 24 | α-Cadinol | 1661 | 1653 | 1.96 | C_15_H_26_O |
| 25 | Caryophyllenol-II | 1680 | 1675 | 2.87 | C_15_H_24_O |
| 26 | Octadecane | 8000 | _ | 0.58 | C_18_H_38_ |
| 27 | Hexahydrofarnesyl acetone | 8041 | 1855 | 1.57 | C_18_H_36_O |
| 28 | n-Hexadecanoic acid= Hexadecanoic acid | 2093 | 1977 | 1.29 | C_16_H_32_O_2_ |
|  | Total |  |  | 99.99 |  |
|  | Monoterpenes hydrocarbons |  |  | 2.27 |  |
|  | Oxygenated monoterpenes |  |  | 21.25 |  |
|  | Sesquiterpenes hydrocarbons |  |  | 25.34 |  |
|  | Oxygenated sesquiterpenes |  |  | 27.5 |  |
|  | Others (Nonterpenoids) |  |  | 23.63 |  |

RI refers to the retention index identified by database NIST 014; RI*refers to the retention index calculated from the retention time relative to that of C8 – C40 n-alkanes

**Table S6- Chemical compounds of *M. Officinalis* leaf essential oil under the effect of silver nanoparticle foliar spraying of 20 mg/L**

| **no.** | **Compound** | **RI^*^** | **RI** | **Mean (%) ± SD** | **Molecular formula** |
| --- | --- | --- | --- | --- | --- |
| 1 | Sulcatone=Methyl heptenone | 984.5 | 965 | 0.27 | C_8_H_14_O |
| 2 | 3-Methyl-2-(2-methyl-2-butenyl)-furan= Rosefuran; α-Naginatene | 1092 | 1095 | 0.36 | C_10_H_14_O |
| 3 | Linalool= Linalol | 1102 | 1097 | 0.31 | C_10_H_18_O |
| 4 | Nonanal= Nonylic aldehyde | 1105 | 1102 | 0.31 | C_9_H_18_O |
| 5 | Photocitral A | 1142 | __ | 0.29 | C_10_H_16_O |
| 6 | 6-Octenal, 7-methyl-3-methylene- | 1144 | 1146.8 | 0.26 | C_10_H_16_O |
| 7 | Citronellal =Citronellal; Rhodinal | 1154 | 1153 | 3.03 | C_10_H_18_O |
| 8 | 3,6-Octadienal, 3,7-dimethyl- | 1181 | 1183.9 | 2.5 | C_10_H_16_O |
| 9 | Citronellol | 1232 | 1259 | 0.87 | C_10_H_20_O |
| 10 | β-Citral | 1246 | 1235 | 10.69 | C_10_H_16_O |
| 11 | methyl ester= Methyl citronellate;; Citronellic acid, methyl ester; | 1258 | 1260 | 1.52 | C_11_H_20_O_2_ |
| 12 | Naphthalene, 1,2,3,4-tetrahydro-1,1,6-trimethyl-= α-Ionene | 1261 | 1258 | 0.34 | C_13_H_18_ |
| 13 | α-Citral; Geranial | 1277 | 1269 | 14.59 | C_10_H_16_O |
| 14 | trans-Geranic acid methyl ester= Geranic acid methyl ester; Methyl geranoate | 1323 | 1321.7 | 2.4 | C_11_H_18_O_2_ |
| 15 | Alpha-Damascone | 1339 | _ | 0.22 | C_13_H_20_O |
| 16 | 2,6-Octadien-1-ol, 3,7-dimethyl- | 1358 | _ | 0.52 | C_10_H_18_O |
| 17 | Geranyl acetate= Acetic acid, geraniol ester, Geraniol acetate | 1381 | 1379 | 10.14 | C_12_H_20_O_2_ |
| 18 | β-Damascenone | 1384 | 1386 | 1.99 | C_13_H_18_O |
| 19 | β-bourbenene | 1392 | 1384 | 2.71 | C_15_H_24_ |
| 20 | β-Elemen | 1395 | 1391 | 0.58 | C_15_H_24_ |
| 21 | Benzene, 1-ethyl-3,5-dimethyl-; 5-Ethyl-1,3-dimethylbenzene | 1400 | 1059 | 1.15 | C_10_H_14_ |
| 22 | Caryophyllene; ; β-Caryophyllen | 1419 | 1451 | 13.33 | C_15_H_24_ |
| 23 | β-Copaene | 1426 | 1428 | 0.32 | C_15_H_24_ |
| 24 | Acetone, geranyl-; Geranyl acetone; Farnesylacetone B; Geranylaceton | 1439 | 1454 | 0.61 | C_13_H_20_O |
| 25 | cis-β-Farnesene= ; β-cis-Farnesene; β-(Z)-Farnesene | 1444 | 1476 | 0.43 | C_15_H_24_ |
| 26 | 2,6,10-Trimethyltridecane | 1453 | 1465.1 | 0.31 | C_16_H_34_ |
| 27 | Humulene= α-Caryophyllene | 1459 | 1453 | 1.05 | C_15_H_24_ |
| 28 | epi-β-Caryophyllene | 1464 | 1467 | 0.52 | C_15_H_24_ |
| 29 | trans-β-Ionone ; β-lonone | 1480 | 1469 | 0.47 | C_13_H_20_O |
| 30 | Germacrene D | 1489 | 1480 | 3.12 | C_15_H_24_ |
| 31 | α-Muurolene | 5005 | - | 0.41 | C_15_H_24_ |
| 32 | Naphthalene, 1,2,3,4,4a,5,6,8a-octahydro-7-methyl-4-methylene-1-(1-methylethyl)-, (1α,4aβ,8aα)- | 5021 | 1513 | 0.2 | C_15_H_24_ |
| 33 | δ-Cadinene, (+)-; (+)-δ-Cadinene | 5025 | 1562 | 1.11 | C_15_H_24_ |
| 34 | (-)-Spathulenol | 5092 | 1582 | 0.7 | C_15_H_24_O |
| 35 | Caryophyllene oxide | 5098 | 1583 | 13.14 | C_15_H_24_O |
| 36 | Hexadecane; Cetane | 6002 | - | 0.36 | C_16_H_34_ |
| 37 | .beta.-Oplopenone: Oplopenone | 1601 | 1607 | 0.3 | C_15_H_24_O |
| 38 | Humulene epoxide 2 | 1611 | 1607 | 0.53 | C_15_H_24_O |
| 39 | 10,10-Dimethyl-2,6-dimethylenebicyclo[7.2.0]undecan-5β-ol | 1639 | 1644.2 | 0.33 | C_15_H_24_O |
| 40 | 11,11-Dimethyl-4,8-dimethylenebicyclo[7.2.0]undecan-3-ol | 1643 | 1645.9 | 1.25 | C_15_H_24_O |
| 41 | α-Cadinol ; (-)-α-Cadinol; l-α-Cadinol; | 1662 | 1653 | 1.62 | C_15_H_26_O |
| 42 | Caryophyllenol-II | 1680 | 1675 | 1.22 | C_15_H_24_O |
| 43 | 2,6,10-Dodecatrienal, 3,7,11-trimethyl-, (E,E)-; Farnesal | 7056 | 1738 | 0.23 | C_15_H_24_O |
| 44 | Octadecane | 8000 | - | 0.29 | C_18_H_38_ |
| 45 | Isopropyl myristate | 8022 | 1823 | 0.31 | C_17_H_34_O_2_ |
| 46 | Hexahydrofarnesyl acetone; Perhydrofarnesyl acetone | 8042 | 1847 | 1.11 | C_18_H_36_O |
| 47 | Cyclotetradecane | 8082 | - | 0.38 | C_14_H_28_ |
| 48 | n-Hexadecanoic acid; Hexadecanoic acid; | 2096 | 1977 | 0.79 | C_16_H_32_O_2_ |
|  | Total |  |  | 98.98 |  |
|  | Monoterpenes hydrocarbons |  |  | 1.15 |  |
|  | Oxygenated monoterpenes |  |  | 33.42 |  |
|  | Sesquiterpenes hydrocarbons |  |  | 23.78 |  |
|  | Oxygenated sesquiterpenes |  |  | 19.32 |  |
|  | Others (Nonterpenoids |  |  | 20.78 |  |

RI refers to the retention index identified by database NIST 014; RI*refers to the retention index calculated from the retention time relative to that of C8 – C40 n-alkanes

**Table S7- Chemical compounds of *M. Officinalis* leaf essential oil under the effect of silver nano particle foliar spraying of 40 mg/L**

| **no.** | **Compound** | **RI^*^** | **RI** | **Mean (%) ± SD** | **Molecular formula** |
| --- | --- | --- | --- | --- | --- |
| 1 | 2-Hexenal, (E)- = Leaf aldehyde t-2-Hexenal | 851.2 | 854 | 0.23 | C_6_H_10_O |
| 2 | 1-Octen-3-ol; Amyl vinyl carbinol;Matsutake alcohol | 981.5 | 980 | 0.38 | C_8_H_16_O |
| 3 | Sulcatone; Prenylacetone | 984.7 | 983 | 0.78 | C_8_H_14_O |
| 4 | β-Myrcene= Myrcene | 990.1 | 990 | 0.24 | C_10_H_16_ |
| 5 | 2,4-Heptadienal, (E,E)-= trans-2-trans-4-Heptadienal; 2,4-Heptadien-1-al; | 1015 | 1011 | 0.25 | C_7_H_10_O |
| 6 | trans-β-Ocimene=trans-Ocimene | 1047 | 1048 | 0.93 | C_10_H_16_ |
| 7 | 3-Methyl-2-(2-methyl-2-butenyl)-furan= ; Rosefuran | 1092 | 1104.3 | 0.34 | C_10_H_14_O |
| 8 | Linalool | 1102 | 1080 | 0.45 | C_10_H_18_O |
| 9 | Nonanal | 1105 | 1102 | 0.22 | C_9_H_18_O |
| 10 | __ | 1109 | _ | 0.11 | _ |
| 11 | Photocitral A | 1142 | __ | 0.45 | C_10_H_16_O |
| 12 | 6-Octenal, 7-methyl-3-methylene- = 7-Methyl-3-methyleneoct-6-enal | 1144 | 1146.8 | 0.35 | C_10_H_16_O |
| 13 | Citronellal= ; Citronellel= ; β-Citronellal; Rhodinal | 1155 | 1170 | 6.05 | C_10_H_18_O |
| 14 | 3,6-Octadienal, 3,7-dimethyl- | 1163 | 1183.9 | 3.43 | C_10_H_16_O |
| 15 | Rose furan oxide; Rosefuran epoxide | 1171 | 1177.2 | 0.23 | C_10_H_14_O_2_ |
| 16 | Citronellol= Cephrol, Rhodinol | 1232 | 1232 | 0.64 | C_10_H_20_O |
| 17 | Neral; β-Citral; Z-Citral | 1246 | 1240 | 17.19 | C_10_H_16_O |
| 18 | Methyl citronellate; Citronellic acid, methyl ester | 1258 | 1260 | 2.58 | C_11_H_20_O_2_ |
| 19 | Geranial; β-Geranial | 1276 | 1269 | 20.78 | C_10_H_16_O |
| 20 | trans-Geranic acid methyl ester؛ Geranic acid methyl ester; Methyl geranoate | 1322 | 1321.7 | 1.57 | C_11_H_18_O_2_ |
| 21 | Nerol acetate; Neryl acetate | 1358 | 1365 | 0.26 | C_12_H_20_O_2_ |
| 22 | Geranyl acetate= Acetic acid, geraniol ester; Geranyl ethanoate | 1379 | 1383 | 6.94 | C_12_H_20_O_2_ |
| 23 | α-Cubebene | 1383 | 1351 | 0.91 | C_15_H_24_O |
| 24 | (-)-β-Bourbonene= β-Bourbonene | 1392 | 1384 | 1.13 | C_15_H_24_O |
| 25 | β-Elemen | 1395 | 1391 | 0.62 | C_15_H_24_O |
| 26 | Benzene, 1-ethyl-2,3-dimethyl-= o-Xylene, 3-ethyl-= 3-Ethyl-o-xylene | 4095 | 1106 | 0.46 | C_10_H_14_ |
| 27 | Caryophyllene =; β-Caryophyllen; β-Caryophyllene | 1417 | 1451 | 10.81 | C_15_H_24_ |
| 28 | β-Copaene | 1426 | 1428 | 0.19 | C_15_H_24_ |
| 29 | cis-β-Farnesene | 1444 | 1476 | 0.27 | C_15_H_24_ |
| 30 | Humulene= ; α-Humullene; (E)-α-Caryophyllene | 1458 | 1453 | 1.16 | C_15_H_24_ |
| 31 | Alloaromadendrene =Alloaromadedrene; allo-aromandendrene | 1463 | 1468 | 0.31 | C_15_H_24_ |
| 32 | Germacrene D | 1488 | 1519 | 3.11 | C_15_H_24_ |
| 33 | Torreyol ; α-Muurolol ; Muurolol | 5004 | 1645 | 0.34 | C_15_H_26_O |
| 34 | γ-Cadinene | 5021 | 1513 | 0.17 | C_15_H_24_ |
| 35 | Naphthalene, 1,2,3,4,4a,5,6,8a-octahydro-7-methyl-4-methylene-1-(1-methylethyl)-, (1α,4aβ,8aα)- : γ-Cadinene | 5024 | 1562 | 0.9 | C_15_H_24_ |
| 36 | (2E,4S,7E)-4-Isopropyl-1,7-dimethylcyclodeca-2,7-dienol= Germacrene D-4-ol | 5088 | 1574 | 0.74 | C_15_H_26_O |
| 37 | Caryophyllene oxide= Caryophylene oxide | 5096 | 1583 | 9.83 | C_15_H_24_O |
| 38 | Humulene-1,2-epoxide; Humulene epoxide 2 | 1610 | 1607 | 0.39 | C_15_H_24_O |
| 39 | 10,10-Dimethyl-2,6-dimethylenebicyclo[7.2.0]undecan-5β-ol Caryophylla-4(12),8(13)-dien-5.beta.-o | 1639 | 1644.2 | 0.2 | C_15_H_24_O |
| 40 | 10,10-Dimethyl-2,6-dimethylenebicyclo[7.2.0]undecan-5β-ol | 1643 | 1644.2 | 0.88 | C_15_H_24_O |
| 41 | α-Cadinol | 1647 | 1653 | 0.31 | C_15_H_26_O |
| 42 | τ-Muurolol | 1661 | 1648 | 1.6 | C_15_H_26_O |
| 43 | _ | 1668 | _ | 0.29 | _ |
| 44 | 2,6,10-Dodecatrienal, 3,7,11-trimethyl-, (E,E)-; farnesyl aldehyde; Farnesal | 7056 | 1738 | 0.18 | C_15_H_24_O |
| 45 | Hexahydrofarnesyl acetone= Perhydrofarnesyl acetone | 8041 | 1847 | 0.43 | C_18_H_36_O |
| 46 | n-Hexadecanoic acid; Hexadecanoic acid;Palmitic acid | 2092 | 1977 | 0.3 | C_16_H_32_O_2_ |
|  | Total |  |  | 68.66 |  |
|  | Monoterpenes hydrocarbons |  |  | 22.41 |  |
|  | Oxygenated monoterpenes |  |  | 11.49 |  |
|  | Sesquiterpenes hydrocarbons |  |  | 19.58 |  |
|  | Oxygenated sesquiterpenes |  |  | 15.76 |  |
|  | Others (Nonterpenoids) |  |  | 6.55 |  |

RI refers to the retention index identified by database NIST 014; RI*refers to the retention index calculated from the retention time relative to that of C8 – C40 n-alkanes

**Table S7- Chemical compounds of *M. Officinalis* leaf essential oil under the influence of silver nano particle foliar spraying of 60 mg/L**

| **no.** | **Compound** | **RI^*^** | **RI** | **Mean (%) ± SD** | **Molecular formula** |
| --- | --- | --- | --- | --- | --- |
| 1 | Nonanal= Nonylaldehyde | 1005 | 1102 | 0.65 | C_9_H_18_O |
| 2 | Citronellal= β-Citronellal; Rhodinal; Citronella; Citronelal | 1153 | 1170 | 2.54 | C_10_H_18_O |
| 3 | 3,6-Octadienal, 3,7-dimethyl- | 1181 | 1183.9 | 2.71 | C_10_H_16_O |
| 4 | 2,6-Octadien-1-ol, 3,7-dimethyl-, (Z)-; cis-Geraniol | 1228 | 1229 | 1.35 | C_10_H_18_O |
| 5 | 2,6-Octadienal, 3,7-dimethyl-, (Z)-= β-Citral, Neroli aldehyde | 1272 | 1235 | 33.05 | C_10_H_16_O |
| 6 | 2,6-Octadien-1-ol, 3,7-dimethyl-, (E)-= Geraniol; Guaniol; Lemonol | 1253 | 1254 | 0.65 | C_10_H_18_O |
| 7 | Methyl citronellate; Citronellic acid, methyl ester | 1257 | 1260 | 1.52 | C_11_H_20_O_2_ |
| 8 | Thymol= Thyme camphor | 1300 | 1294 | 1.7 | C_10_H_14_O |
| 9 | Carvacrol; Antioxine | 1308 | 1302 | 1.27 | C_10_H_14_O |
| 10 | trans-Geranic acid methyl ester=Geranic acid methyl ester; Methyl geranoate | 1322 | 1324 | 2.82 | C_11_H_18_O_2_ |
| 11 | Geranyl acetate= Acetic acid, geraniol ester | 1378 | 1383 | 10.81 | C_12_H_20_O_2_ |
| 12 | Damascenone | 1383 | 1386 | 2.17 | C_13_H_18_O |
| 13 | 5-Amino-1-phenylpyrazole | 1391 | _ | 1.77 | C_9_H_9_N_3_ |
| 14 | Tetradecane | 4001 | _ | 0.33 | C_14_H_30_ |
| 15 | Benzene, 1,2,4,5-tetramethyl-= Durene | 4092 | _ | 2.15 | C_10_H_14_ |
| 16 | Caryophyllene= β-cariofillene; β-caryophellene | 1415 | 1451 | 11.82 | C_15_H_24_ |
| 17 | Geranyl acetone | 1437 | 1454 | 0.44 | C_13_H_22_O |
| 18 | 2,6,10-Trimethyltridecane | 1452 | 1465 | 0.93 | C_16_H_34_ |
| 19 | Humulene : α-Caryophyllene | 1458 | 1488 | 1.11 | C_15_H_24_ |
| 20 | epi-β-Caryophyllene | 1463 | 1467 | 1.76 | C_15_H_24_ |
| 21 | Pentadecane | 5000 | _ | 0.63 | C_15_H_24_ |
| 22 | Caryophyllene oxide | 5094 | 1583 | 11.15 | C_15_H_24_O |
| 23 | Hexadecane= Cetane | 6000 | _ | 0.98 | C_16_H_34_ |
| 24 | 10,10-Dimethyl-2,6-dimethylenebicyclo[7.2.0]undecan-5β-ol | 1643 | 1644.2 | 1.23 | C_15_H_24_O |
| 25 | Caryophyllenol-II | 1680 | 1676 | 0.98 | C_15_H_24_O |
| 26 | Octadecane= Octadecan | 8000 | _ | 0.45 | C_18_H_38_ |
| 27 | Perhydrofarnesyl acetone | 8041 | 1847 | 2.03 | C_18_H_36_O |
| 28 | n-Hexadecanoic acid= Hexadecanoic acid; Palmitic acid; Palmitinic acid; | 2092 | 1971 | 1.00 | C_16_H_32_O_2_ |
|  | Total |  |  | 100 |  |
|  | Monoterpenes hydrocarbons |  |  | 2.15 |  |
|  | Oxygenated monoterpenes |  |  | 43.27 |  |
|  | Sesquiterpenes hydrocarbons |  |  | 15.32 |  |
|  | Oxygenated sesquiterpenes |  |  | 13.36 |  |
|  | Others (Nonterpenoids) |  |  | 25.9 |  |

RI refers to the retention index identified by database NIST 014; RI*refers to the retention index calculated from the retention time relative to that of C8 – C40 n-alkanes

**Table S8- Chemical compounds of *M. Officinalis* leaf essential oil under the influence of silver nano particle foliar spraying of 80 mg/L**

| **no.** | **Compound** | **RI^*^** | **RI** | **Mean (%) ± SD** | **Molecular formula** |
| --- | --- | --- | --- | --- | --- |
| 1 | Acetonyldimethylcarbinol; Diacetone alcohol; Diketone alcohol A | 838.3 | 830 | 0.33 | C_6_H_12_O_2_ |
| 2 | Methyl heptenone; Sulcatone | 984.4 | _ | 0.57 | C_8_H_14_O |
| 3 | Linalool | 1102 | 1080 | 0.5 | C_10_H_18_O |
| 4 | Nonanal | 1105 | 1102 | 0.48 | C_9_H_18_O |
| 5 | Cyclohexene, 3-isobutyl | 1142 | __ | 0.47 | C_10_H_18_ |
| 6 | 6-Octenal, 7-methyl-3-methylene- | 1144 | 1146.8 | 0.42 | C_10_H_16_O |
| 7 | Citronellal= β-Citronellal; Rhodinal | 1153 | 1154 | 4.71 | C_10_H_18_O |
| 8 | 3,6-Octadienal, 3,7-dimethyl- | 1181 | 1183.9 | 4.06 | C_10_H_16_O |
| 9 | Cinerin I; Cinerin | 1212 | _ | 0.46 | C_20_H_28_O_3_ |
| 10 | cis-Geraniol | 1254 | 1229 | 23.6 | C_10_H_18_O |
| 11 | 6-Octenoic acid, 3,7-dimethyl-, methyl ester= Methyl citronellate; Citronellic acid, methyl ester | 1257 | 1260 | 1.7 | C_11_H_20_O_2_ |
| 12 | Citral | 1273 | _ | 28.53 | C_10_H_16_O |
| 13 | Tridecane | 1300 | _ | 0.54 | C_13_H_28_ |
| 14 | trans-Geranic acid methyl ester=Geranic acid methyl ester; Methyl geranoate | 1322 | 1321.7 | 2.66 | C_11_H_18_O_2_ |
| 15 | Geranyl acetate= Acetic acid, geraniol ester; Geraniol acetate | 1377 | 1383 | 8.33 | C_12_H_20_O_2_ |
| 16 | 2-Buten-1-one, 1-(2,6,6-trimethyl-1,3-cyclohexadien-1-yl)-, (E)-=Damascenone | 1383 | 1386 | 1.17 | C_13_H_18_O |
| 17 | 5-Amino-3-phenylpyrazole | 1391 | _ | 0.85 | C_9_H_9_N_3_ |
| 18 | Benzene, 1-ethyl-3,5-dimethyl- | 4092 | _ | 0.99 | C_10_H_14_ |
| 19 | Caryophyllene= β-Caryophyllen | 1414 | 1420 | 4.92 | C_15_H_24_ |
| 20 | Humulene; α-Caryophyllene | 1457 | 1453 | 0.53 | C_15_H_24_ |
| 21 | Aromandendrene | 1463 | 1440 | 0.66 | C_15_H_24_ |
| 22 | Pentadecane | 5000 | _ | 0.31 | C_15_H_32_ |
| 23 | (-)-Spathulenol | 5088 | 1582 | 0.36 | C_15_H_24_O |
| 24 | Caryophyllene oxide; Caryophylene oxide | 5094 | 1583 | 9.42 | C_15_H_24_O |
| 25 | Hexadecane= Cetane | 5099 | _ | 0.6 | C_16_H_34_ |
| 26 | (1R,3E,7E,11R)-1,5,5,8-Tetramethyl-1,2-oxabicyclo[9.1.0]dodeca-3,7-diene= Humulene oxide II; | 1610 | 1607 | 0.37 | C_15_H_24_O |
| 27 | Caryophylla-4(12),8(13)-dien-5α-ol= Caryophylladienol II | 1643 | 1678 | 0.85 | C_15_H_24_O |
| 28 | 2-Pentadecanone, 6,10,14-trimethyl-; Hexahydrofarnesyl acetone;; | 8041 | 1847 | 1.09 | C_18_H_36_O |
| 29 | n-Hexadecanoic acid; Hexadecanoic acid: Palmitic acid | 2091 | 1977 | 0.5 | C_16_H_32_O_2_ |
|  | Total |  |  | 98.98 |  |
|  | Monoterpenes hydrocarbons |  |  | 1.46 |  |
|  | Oxygenated monoterpenes |  |  | 61.82 |  |
|  | Sesquiterpenes hydrocarbons |  |  | 6.42 |  |
|  | Oxygenated sesquiterpenes |  |  | 11 |  |
|  | Others (Nonterpenoids |  |  | 18.28 |  |

RI refers to the retention index identified by database NIST 014; RI*refers to the retention index calculated from the retention time relative to that of C8 – C40 n-alkanes

**Table S9- Chemical compounds of *M. Officinalis* leaf essential oil under the effect of silver nano particle foliar spraying of 100 mg/L**

| **no.** | **Compound** | **RI^*^** | **RI** | **Mean (%) ± SD** | **Molecular formula** |
| --- | --- | --- | --- | --- | --- |
| 1 | Leaf aldehyde | 851.2 | 854 | 0.33 | C_6_H_10_O |
| 2 | Prenylacetone | 984.4 | 965 | 0.37 | C_8_H_14_O |
| 3 | 2,4-Heptadienal, (E,E)- | 1013 | 1007 | 0.38 | C_7_H_10_O |
| 4 | 1,3,6-Octatriene, 3,7-dimethyl-, (Z)- | 1047 | 1039 | 0.41 | C_10_H_16_ |
| 5 | Linalool | 1102 | 1100 | 0.32 | C_10_H_18_O |
| 6 | Nonanal =Pelargonic aldehyde | 1105 | 1102 | 0.56 | C_9_H_18_O |
| 7 | 6-Octenal, 7-methyl-3-methylene- | 1144 | 1146.8 | 0.28 | C_10_H_16_O |
| 8 | Citronellal | 1154 | 1170 | 3.23 | C_10_H_18_O |
| 9 | 3,6-Octadienal, 3,7-dimethyl- | 1181 | 1183.9 | 2.79 | C_10_H_16_O |
| 10 | (Z)-Geraniol; Nerol; Neryl alcohol | 1229 | 1229 | 0.64 | C_10_H_18_O |
| 11 | β-Citral; | 1244 | 1235 | 13.35 | C_10_H_16_O |
| 12 | Geraniol; Lemonol | 1254 | 1250 | 0.48 | C_10_H_18_O |
| 13 | Methyl citronellate | 1257 | 1260 | 1.46 | C_11_H_20_O_2_ |
| 14 | Citral | 1274 | - | 17.65 | C_10_H_16_O |
| 15 | Thymol | 1300 | 1294 | 0.4 | C_10_H_14_O |
| 16 | trans-Geranic acid methyl ester =; Geranic acid methyl ester; Methyl geranoate | 1322 | 1321.7 | 2.09 | C_11_H_18_O_2_ |
| 17 | 2-Buten-1-one, 1-(2,6,6-trimethyl-2-cyclohexen-1-yl)-, (E)- | 1338 | - | 0.27 | C_13_H_20_O |
| 18 | Geranyl acetate; Acetic acid, geraniol ester | 1379 | 1376 | 10.47 | C_12_H_20_O_2_ |
| 19 | β-Damascenone; Damascenone | 1383 | 1386 | 1.8 | C_13_H_18_O |
| 20 | 5-Amino-1-phenylpyrazole | 1391 | - | 1.36 | C_9_H_9_N_3_ |
| 21 | Benzene, 1,2,3,5-tetramethyl- | 1494 | 1115 | 2.29 | C_10_H_14_ |
| 22 | Caryophyllene | 1415 | 1451 | 7.07 | C_15_H_24_ |
| 23 | cis-Geranylacetone; Nerylacetone | 1438 | 1434 | 0.41 | C_13_H_22_O |
| 24 | 2,6,10-Trimethyltridecane | 1452 | 1465 | 0.5 | C_16_H_34_ |
| 25 | Humulene = α-Caryophyllene; | 1458 | 1488 | 1.09 | C_15_H_24_ |
| 26 | Alloaromadendrene | 1463 | 1458 | 0.94 | C_15_H_24_ |
| 27 | 3-Buten-2-one, 4-(2,6,6-trimethyl-1-cyclohexen-1-yl)- =β-Ionone; β-Cyclocitrylideneacetone | 1479 | 1488.4 | 0.32 | C_13_H_20_O |
| 28 | Germacrene D | 1488 | 1519 | 2.2 | C_15_H_24_ |
| 29 | Pentadecane | 5000 | - | 048 | C_15_H_24_ |
| 30 | Naphthalene, 1,2,3,5,6,8a-hexahydro-4,7-dimethyl-1-(1-methylethyl)-, (1S-cis)- | 5040 |  | 0.77 | C_15_H_24_ |
| 31 | Naphthalene, 1,6,7-trimethyl- | 5061 | 1572 | 1.14 | C_13_H_14_ |
| 32 | Naphthalene, 2,3,6-trimethyl- | 5056 | - | 0.39 | C_13_H_14_ |
| 33 | (-)-Spathulenol | 5089 | 1582 | 0.53 | C_15_H_24_O |
| 34 | Caryophyllene oxide | 5095 | 1583 | 11.54 | C_15_H_24_O |
| 35 | Hexadecane= Cetane | 6001 | - | 1.43 | C_16_H_34_ |
| 36 | Humulene epoxide II | 1610 | 1607 | 0.49 | C_15_H_24_O |
| 37 | 10,10-Dimethyl-2,6-dimethylenebicyclo[7.2.0]undecan-5β-ol | 1639 | 1644.2 | 0.63 | C_15_H_24_O |
| 38 | 11,11-Dimethyl-4,8-dimethylenebicyclo[7.2.0]undecan-3-ol | 1643 | 1645.9 | 1.54 | C_15_H_24_O |
| 39 | α-Cadinol | 1661 | 1653 | 1.86 | C_15_H_26_O |
| 40 | Naphthalene, decahydro-4a-methyl-1-methylene-7-(1-methylethenyl)-, [4aR-(4aα,7α,8aβ)]- | 1680 | 1490 | 1.41 | C_15_H_24_ |
| 41 | Humulane-1,6-dien-3-ol | 1696 | - | 0.4 | C_15_H_26_O |
| 42 | Heptadecane | 7000 | - | 0.43 | C_17_H_36_ |
| 43 | Octadecane | 8000 | - | 0.59 | C_18_H_38_ |
| 44 | 2-Pentadecanone, 6,10,14-trimethyl- | 8041 | 1847 | 1.21 | C_18_H_36_O |
| 45 | Phthalic acid, 7-bromoheptyl isobutyl ester | 8061 | ---- | 0.34 | C_19_H_27_ BrO_4_ |
| 46 | 1-Methyldibenzothiophene | 8070 | 319.22 | 0.33 | C_13_H_10_S |
| 47 | n-Hexadecanoic acid =Hexadecanoic acid =Palmitic acid | 2094 | 1977 | 1.06 | C_16_H_32_O_2_ |
|  | Total |  |  | 100.03 |  |
|  | Monoterpenes hydrocarbons |  |  | 2.7 |  |
|  | Oxygenated monoterpenes |  |  | 40.6 |  |
|  | Sesquiterpenes hydrocarbons |  |  | 13.96 |  |
|  | Oxygenated sesquiterpenes |  |  | 16.99 |  |
|  | Others (Nonterpenoids) |  |  | 25.78 |  |

RI refers to the retention index identified by database NIST 014; RI*refers to the retention index calculated from the retention time relative to that of C8 – C40 n-alkanes

**Table S10- Chemical compounds of *M. Officinalis* leaf essential oil under the influence of control foliar application**

| **no.** | **Compound** | **RI^*^** | **RI** | **Mean (%) ± SD** | **Molecular formula** |
| --- | --- | --- | --- | --- | --- |
| 1 | 2-Hexenal, (E)- | 851.1 | 854 | 0.47 | C_6_H_10_O |
| 2 | Sulcatone, Prenylacetone | 987.02 | 993 | 3.21 | C_8_H_14_O |
| 3 | D-Limonene | 1032 | - | 0.3 | C_10_H_16_ |
| 4 | Melonal | 1054 | - | 0.3 | C_9_H_16_O |
| 5 | furan | 1093 | 1104.3 | 0.96 | C_10_H_14_O |
| 6 | Perillen | 1100 | 1102.1 | 0.24 | C_10_H_14_O |
| 7 | Linalool= Phantol | 1103 | 1080 | 0.63 | C_10_H_18_O |
| 8 | Photpcitral A | 1143 |  | 0.49 | C_10_H_16_O |
| 9 | 6-Octenal, 7-methyl-3-methylene- | 1145 | 1146.8 | 0.65 | C_10_H_16_O |
| 10 | Citronellal= Citronella | 1156 | 1170 | 4.54 | C_10_H_18_O |
| 11 | 3,6-Octadienal, 3,7-dimethyl- =3,7-Dimethyl-3,6-octadienal | 1183 | 1183.9 | 5.33 | C_10_H_16_O |
| 12 | Furan, Rosefuran epoxide | 1172 | 1177.2 | 1.4 | C_10_H_14_O_2_ |
| 13 | β-Citral | 1253 | 1235.0 | 24.47 | C_10_H_16_O |
| 14 | Methyl citronellate =methyl ester, | 1260 | 1260. | 0.61 | C_11_H_20_O_2_ |
| 15 | 2-Cyclohexen-1-one,3-methyl-6-(1-methylethyl)- =Piperitone, | 1264 | 1268 | 0.59 | C_10_H_16_O |
| 16 | α-Citral= Geranial | 1286 | 1269. | 28.25 | C_10_H_16_O |
| 17 | trans-Geranic acid methyl ester=3,7-dimethyl , Methyl geranoate | 1324 | 1321.7 | 1.1 | C_11_H_18_O_2_ |
| 18 | Geranyl acetate | 1382 | 1379 | 5.99 | C_12_H_20_O_2_ |
| 19 | α-Cubebene | 1384 | 1351. | 0.51 | C_15_H_24_ |
| 20 | (-)-β-Bourbonene | 1393 | 1386 | 0.47 | C_15_H_24_ |
| 21 | Caryophyllene | 1417 | 1451 | 3.36 | C_15_H_24_ |
| 22 | Humulene | 1459 | 1456 | 0.36 | C_15_H_24_ |
| 23 | Aromandendrene | 1464 | 1440 | 0.47 | C_15_H_24_ |
| 24 | aromatic curcumene | 1484 | 1493 | 2.61 | C_15_H_24_ |
| 25 | δ-Cadinene | 5025 | 1562 | 0.5 | C_15_H_24_ |
| 26 | 1,6,10-Dodecatrien-3-ol, 3, 7, 11-trimethyl- | 5066 | 1565 | 0.53 | C_15_H_26_O |
| 27 | ar-Tumerol | 5085 | - | 0.32 | C_15_H_22_O |
| 28 | Espatulenol | 5093 | 1622 | 2.09 | C_15_H_24_O |
| 29 | Caryophyllene oxide | 5098 | 1589 | 7.36 | C_15_H_24_O |
| 30 | Humulene epoxide 2 | 1611 | 1607 | 0.52 | C_15_H_24_O |
| 31 | Bicyclo[4.4.0]dec-1-ene, 2-isopropyl-5-methyl-9-methylene- | 1646 | - | 0.54 | C_15_H_24_ |
| 32 | Caryophyllenol-II | 1680 | 1675 | 0.57 | C_15_H_24_O |
| 33 | Perhydrofarnesyl acetone | 8041 | 1855 | 0.27 | C_18_H_36_O |
|  | **Total** |  |  | 100.01 |  |
|  | Monoterpenes hydrocarbons |  |  | 0.3 |  |
|  | Oxygenated monoterpenes |  |  | 68.04 |  |
|  | Sesquiterpenes hydrocarbons |  |  | 8.82 |  |
|  | Oxygenated sesquiterpenes |  |  | 11.39 |  |
|  | Others (Nonterpenoids) |  |  | 11.46 |  |

RI refers to the retention index identified by database NIST 014; RI*refers to the retention index calculated from the retention time relative to that of C8 – C40 n-alkanes


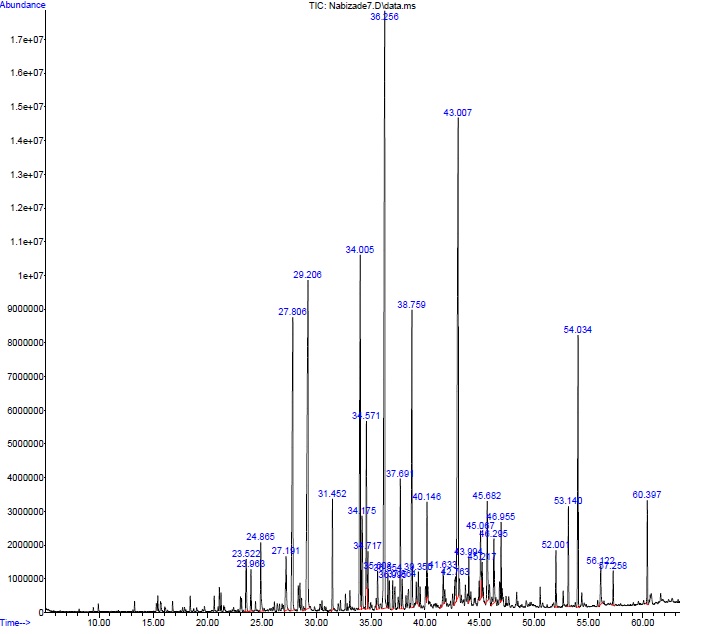


**Figure S1- Chromatogram of the sample sprayed with iron nanoparticles 20 mg/L**

**
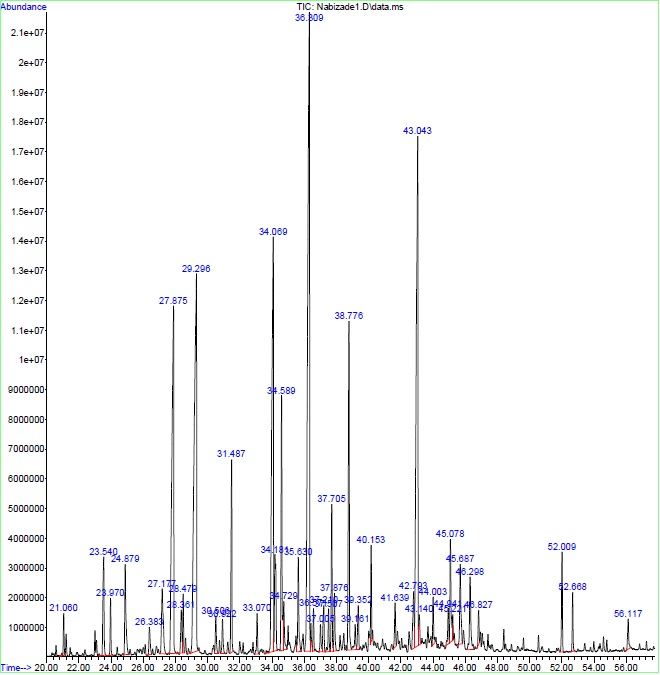
**

**Figure S2- Chromatogram of the sample sprayed with iron nanoparticles 40 mg/L**

**
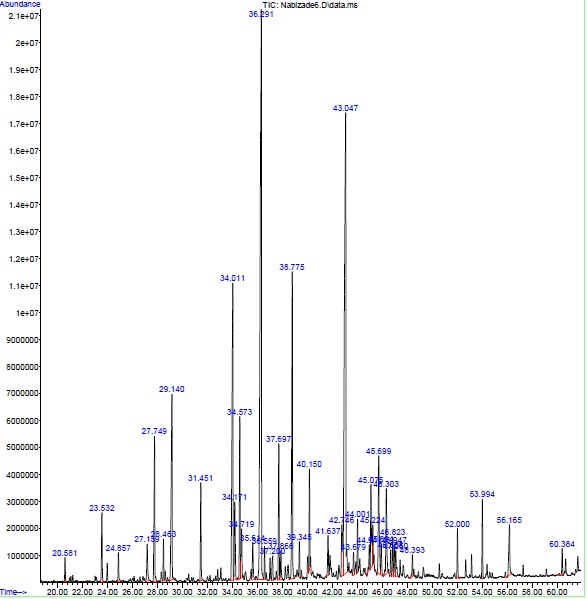
**

**Figure S3-Chromatogram of the sample sprayed with iron nanoparticles 60 mg/L**

**
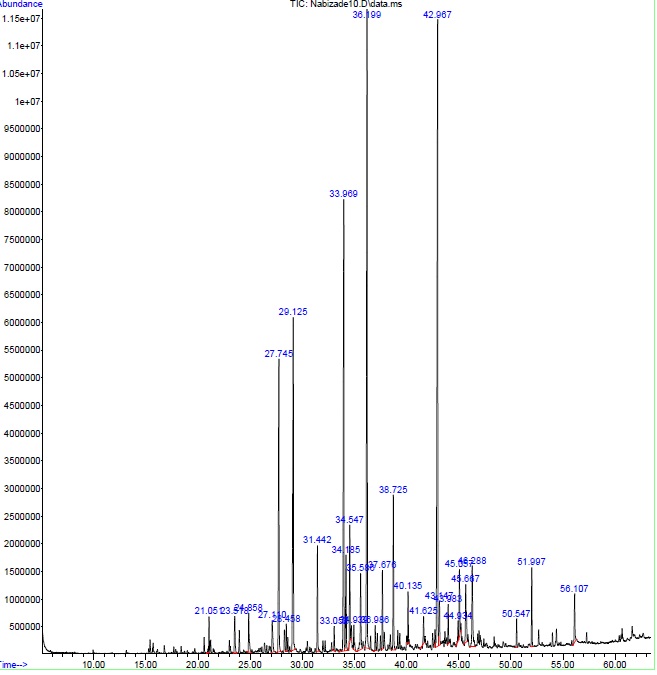
**

**Figure S4- Chromatogram of the sample sprayed with iron nanoparticles 80 mg/L**

**
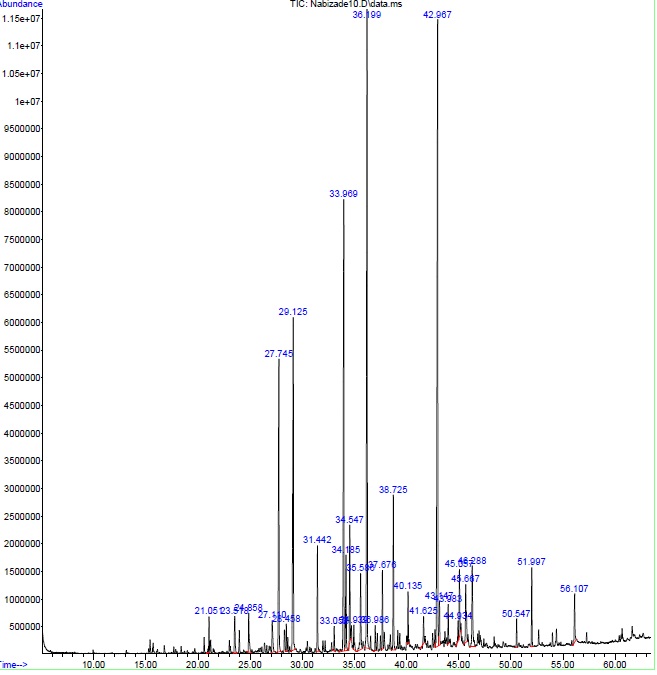
**

**Figure S5- Chromatogram of the sample sprayed with iron nanoparticles 100 mg/L**

**
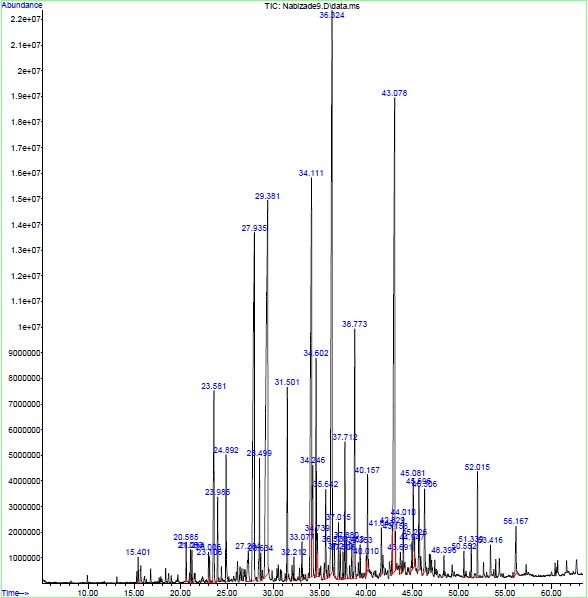
**

**Figure S6- Chromatogram of the sample sprayed with silver nanoparticles 20 mg/L**

**
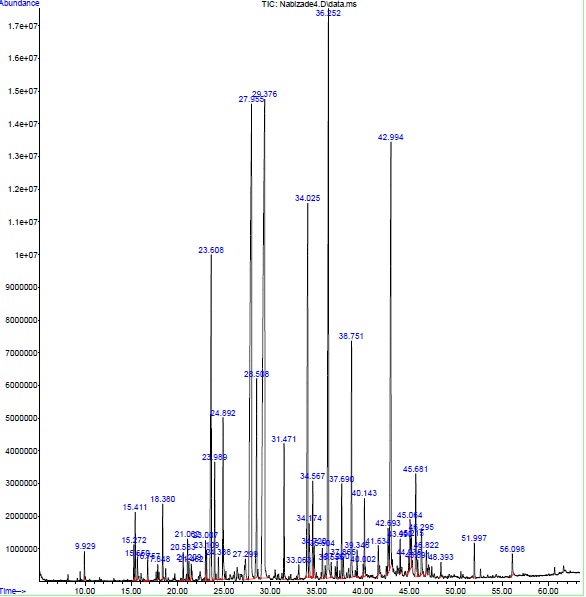
**

**Figure S7- Chromatogram of the sample sprayed with silver nanoparticles 40 mg/L**

**
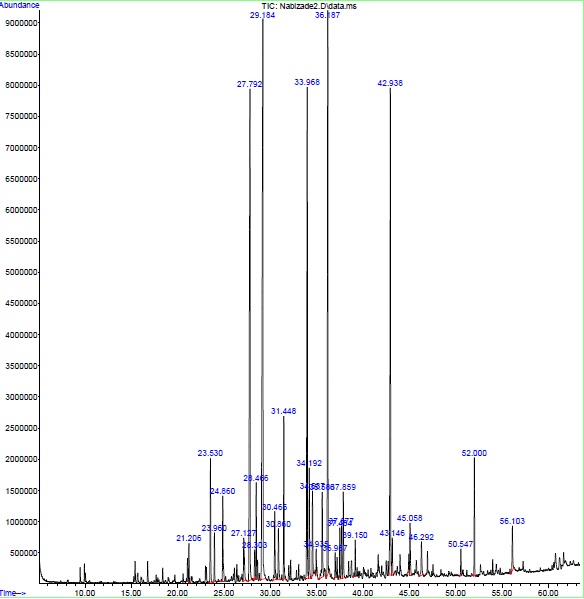
**

**Figure S8-Chromatogram of the sample sprayed with silver nanoparticles 60 mg/L**

**
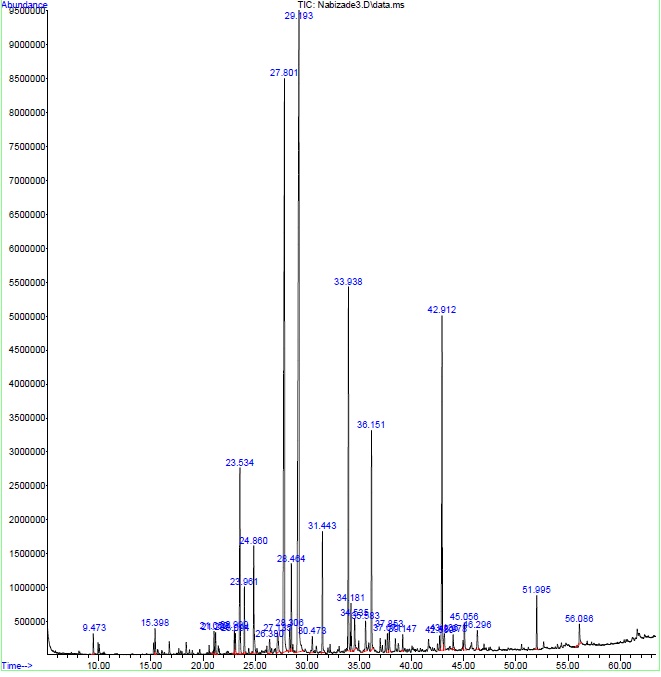
Figure S9- Chromatogram of the sample sprayed with silver nanoparticles 80 mg/L**

**
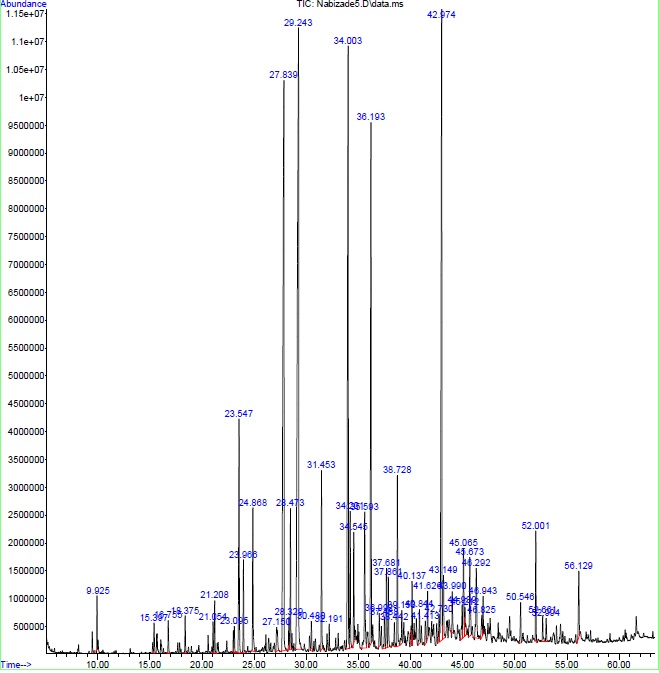
**

**Figure S10-Chromatogram of the sample sprayed with silver nanoparticles 100 mg/L**

**
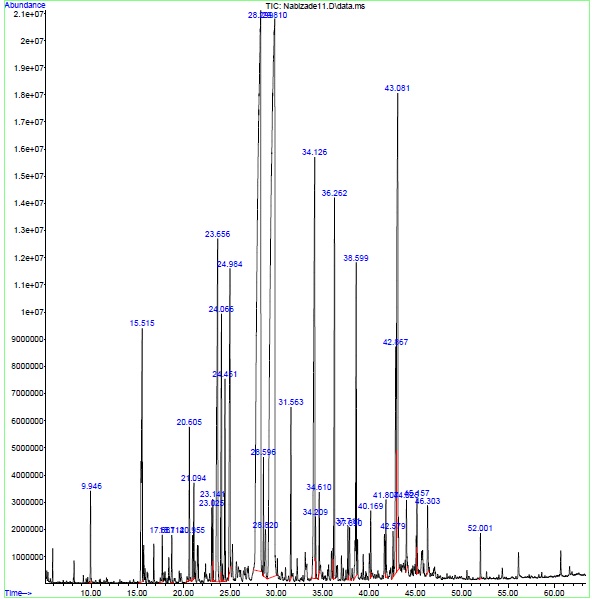
**

**Figure S11-Chromatogram of the sample without foliar application (control**


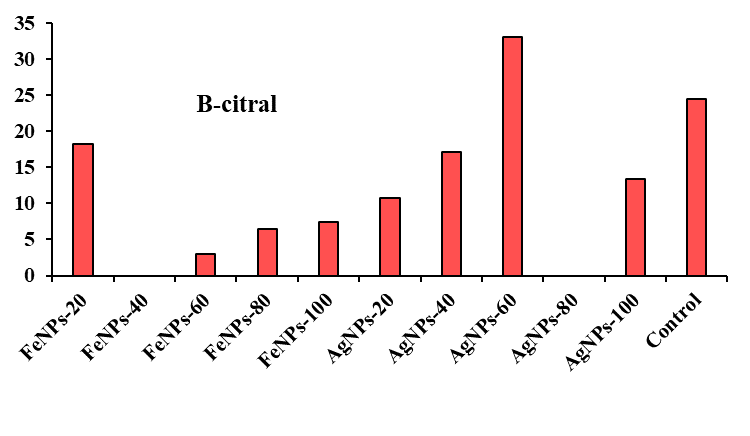


**Figure S12-The amount of beta-citral compound in the essential oil of *M. officinalis* leaves under different treatments**


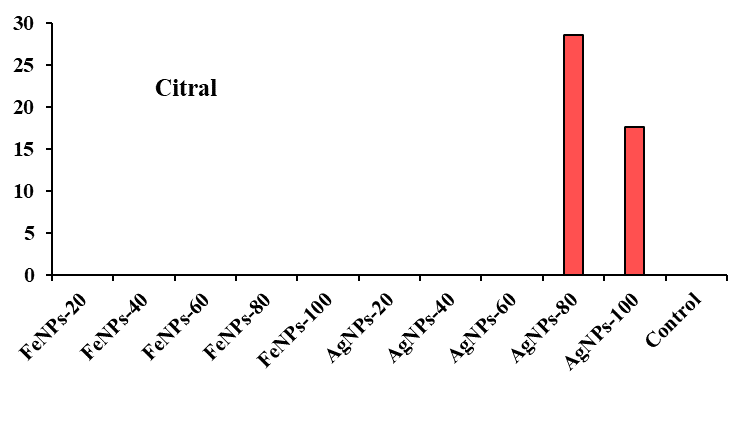


Figure S13-The amount of citral compound in the essential oil of *M. officinalis* leaves under different treatments

**
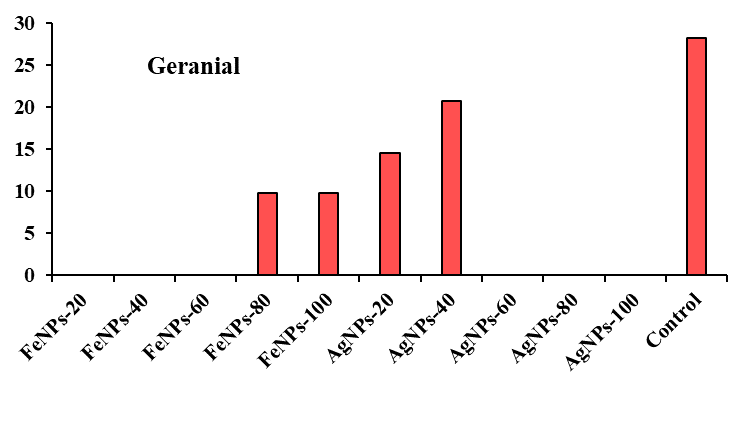
**

**Figure S14-Amount of geranial compound in *M. officinalis* leaf essential oil under different treatments**


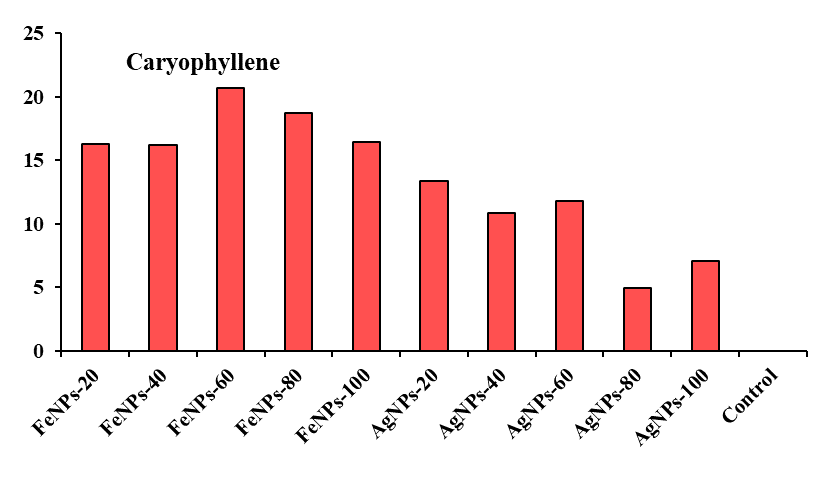


**Figure S15-Amount of caryophyllene composition in *M. officinalis* leaf essential oil under different treatments**


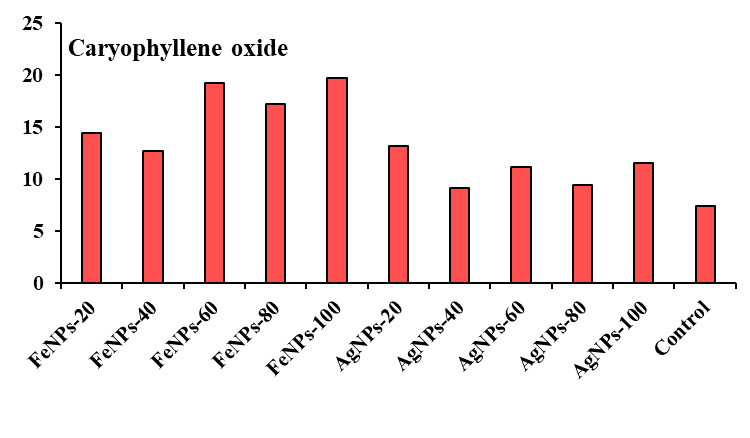


**Figure S16-Amount of caryophyllene oxide compound in *M. officinalis* leaf essential oil under different treatments**

**
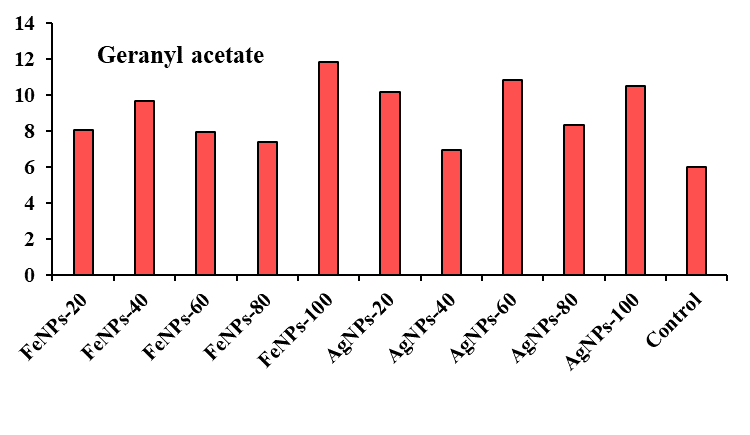
**

**Figure S17-Amount of geranial acetate composition in *M. officinalis* leaf essential oil under different treatment**
